# Molecular basis for the PAM expansion and fidelity enhancement of an evolved Cas9 nuclease

**DOI:** 10.1371/journal.pbio.3000496

**Published:** 2019-10-11

**Authors:** Weizhong Chen, Hongyuan Zhang, Yifei Zhang, Yu Wang, Jianhua Gan, Quanjiang Ji

**Affiliations:** 1 School of Physical Science and Technology, ShanghaiTech University, Shanghai, China; 2 State Key Laboratory of Genetic Engineering, Collaborative Innovation Center of Genetics and Development, Shanghai Public Health Clinical Center, School of Life Sciences, Fudan University, Shanghai, China; IMBA, AUSTRIA

## Abstract

Clustered regularly interspaced short palindromic repeats (CRISPR)-Cas systems have been harnessed as powerful genome editing tools in diverse organisms. However, the off-target effects and the protospacer adjacent motif (PAM) compatibility restrict the therapeutic applications of these systems. Recently, a *Streptococcus pyogenes* Cas9 (SpCas9) variant, xCas9, was evolved to possess both broad PAM compatibility and high DNA fidelity. Through determination of multiple xCas9 structures, which are all in complex with single-guide RNA (sgRNA) and double-stranded DNA containing different PAM sequences (TGG, CGG, TGA, and TGC), we decipher the molecular mechanisms of the PAM expansion and fidelity enhancement of xCas9. xCas9 follows a unique two-mode PAM recognition mechanism. For non-NGG PAM recognition, xCas9 triggers a notable structural rearrangement in the DNA recognition domains and a rotation in the key PAM-interacting residue R1335; such mechanism has not been observed in the wild-type (WT) SpCas9. For NGG PAM recognition, xCas9 applies a strategy similar to WT SpCas9. Moreover, biochemical and cell-based genome editing experiments pinpointed the critical roles of the E1219V mutation for PAM expansion and the R324L, S409I, and M694I mutations for fidelity enhancement. The molecular-level characterizations of the xCas9 nuclease provide critical insights into the mechanisms of the PAM expansion and fidelity enhancement of xCas9 and could further facilitate the engineering of SpCas9 and other Cas9 orthologs.

## Abstract 2

The crystal structures of an evolved Cas9 nuclease, xCas9, in complex with different protospacer adjacent motifs (PAMs) revealed the molecular mechanism for its expanded PAM compatibility and enhanced DNA fidelity.

## Introduction

Clustered regularly interspaced short palindromic repeats (CRISPR)-Cas9 systems, originally discovered in prokaryotic immune systems, have been harnessed and engineered for robust genome editing in diverse organisms [1–8]. When the RNA-guided Cas9 endonuclease is in complex with a mature CRISPR RNA (crRNA) and a transactivating crRNA (tracrRNA) or a chimeric single-guide RNA (sgRNA), Cas9 is capable of cleaving any genomic locus in a programmable manner when a protospacer adjacent motif (PAM) is present [7–10]. The ability to generate site-specific double-stranded DNA breaks via an easily programmable 20-nt guide sequence within the sgRNA renders Cas9 a versatile tool for genetic manipulations in diverse organisms [8,11–16].

Structural studies have provided mechanistic insights into the PAM recognition and cleavage activity of Cas9 [10,17–20]. The most widely utilized Cas9 nuclease from *Streptococcus pyogenes* consists of a nuclease (NUC) lobe and an α-helical recognition (REC) lobe [10,19]. The HNH-like nuclease (HNH) and RuvC-like nuclease (RuvC) domains in the NUC lobe cleave the target DNA strand (complementary to the guide RNA) and the nontarget DNA strand, respectively, while the C-terminal domain (CTD) is involved in the PAM interaction and contributes to the DNA target specificity. The REC lobe is composed of three REC domains (REC1-3) and plays a role in the recognition of guide RNA–target DNA heteroduplexes and cognate sgRNA scaffolds. The binding of a guide RNA to apo-Cas9 triggered a dramatic conformational change, from an open conformation to a cleavage-competent state, leading Cas9 to engage its DNA target [17,18].

In recent years, the *Streptococcus pyogenes* Cas9 (SpCas9) nuclease, which recognizes a 5ʹ-NGG-3ʹ PAM sequence, has been harnessed for applications in a broad range of research, such as genome editing, transcriptional repression and activation, epigenetic modifications, single base-pair conversion, and genomic imaging, in various organisms and cell types [6,21–26]. However, the utility of SpCas9 in therapeutic applications is restricted by its PAM compatibility as well as by its off-target effects [27,28].

To overcome the inherent limitations of wild-type (WT) SpCas9, several natural CRISPR nucleases with different PAM requirements were developed as alternative tools, including *Staphylococcus aureus* Cas9 [29,30], *Neisseria meningitides* Cas9 [31,32], *Francisella novicida* Cas9 [33], *Campylobacter jejuni* Cas9 [34,35], *Acidaminococcus* sp. Cpf1 [36,37], and *Lachnospiraceae bacterium* Cpf1 [36,38]. In addition, a few SpCas9 variants have been engineered. For instance, the VQR (D1135V/R1335Q/T1337R), EQR (D1135E/R1335Q/T1337R), and VRER (D1135V/G1218R/R1335E/T1337R) variants, which recognize 5ʹ-NGAN-3ʹ, 5ʹ-NGNG-3ʹ, and 5ʹ-NGCG-3ʹ PAMs, respectively, were developed by utilizing bacterial selection-based directed evolution [39–41]. Subsequently, SpCas9-NG was rationally engineered to recognize relaxed 5ʹ-NG-3ʹ PAMs by introducing non–base-specific interactions with the PAM duplex to compensate for the loss of base-specific interactions [42]. In addition, several high-fidelity SpCas9 variants, such as SpCas9-HF1, eSpCas9, HypaCas9, and evoCas9, were developed through the mutation of multiple residues involved in RNA–DNA heteroduplex recognition [43–46]. Recently, the SpCas9 variant xCas9, which harbors A262T/R324L/S409I/E480K/E543D/M694I/E1219V mutations, was evolved to possess broad PAM compatibility (5ʹ-NG-3ʹ, 5ʹ-GAA-3ʹ, and 5ʹ-GAT-3ʹ) and substantially lower genome-wide off-target activity than WT SpCas9 [47].

More recently, the structures of xCas9 in complex with sgRNA and 5ʹ-GAT-3ʹ or 5ʹ-AAG-3ʹ PAM-containing DNA were determined, providing certain insights into the molecular mechanisms underlying PAM expansion and fidelity enhancement [48]. It identified that the mutation of E1219V is the structural determinant for the loosened specificity of PAM recognition by xCas9 and revealed that the mutations in the REC domain could enhance the DNA targeting specificity of SpCas9 [48]. Here, we report the crystal structures of xCas9 in complex with four different PAMs (TGG, CGG, TGA, and TGC PAMs). In consistent with the previous work [48], our structures and biochemical assays demonstrated the important role of E1219V mutation in PAM expansion and pinpointed the crucial residues responsible for the fidelity enhancement of xCas9. Intriguingly, our study revealed that xCas9 adopts a unique two-mode PAM recognition mechanism. xCas9 utilizes the same strategy as WT SpCas9 to recognize canonical NGG PAMs. However, xCas9 undergoes striking conformation rearrangement in the REC domain and PAM recognition site when it recognizes non-NGG PAM DNA substrates. In addition, our structures exhibit notable conformational differences in the REC lobe and PAM recognition site compared with the reported xCas9 structures [48], suggesting that xCas9 behaves more flexibly than WT SpCas9 and it can adopt different conformations when recognizing different PAM sequences. The structural and biochemical results presented here provide intensive insights into the mechanisms of the broadened PAM compatibility and improved targeting fidelity of xCas9 and could facilitate the rational engineering of Cas9 orthologs.

## Results

### Determination of xCas9 activity

**Fig 1:**
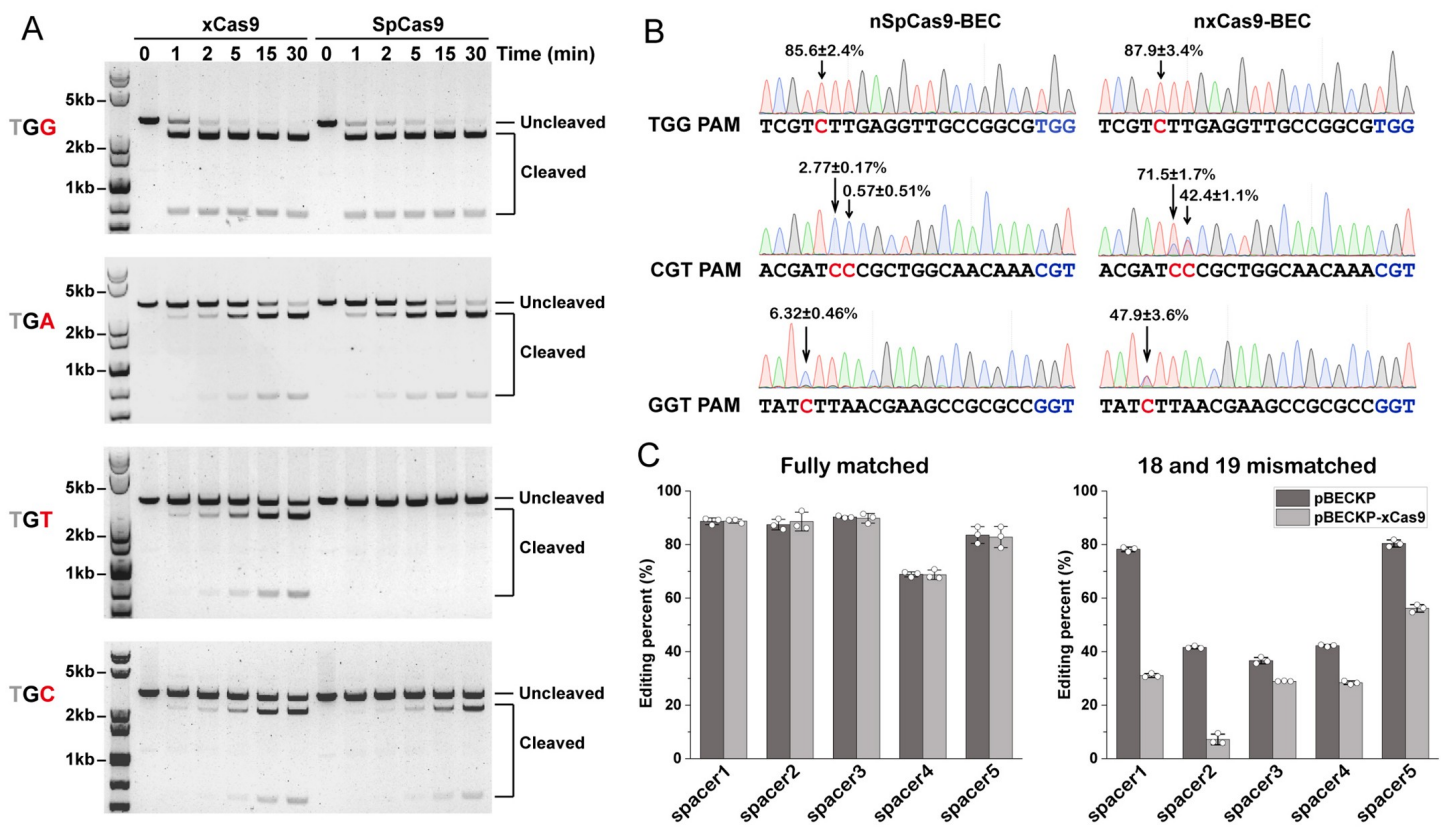
In vitro and vivo activity of xCas9 compared to WT SpCas9. (A) In vitro cleavage assays of xCas9 and WT SpCas9 using linearized plasmids containing a target sequence adjacent to TGG, TGA, TGT, and TGC PAMs. (B) Cytosine base editing efficiency of pBECKP and pBECKP-xCas9 plasmids toward target sequence adjacent to different PAMs in the *K*. *pneumoniae* strain KP_1.6366. The experiments were performed in triplicate, and one representative result was shown. The data underlying this figure can be found in S1 Data. (C) Cytosine base editing efficiency of pBECKP and pBECKP-xCas9 plasmids towards five different spacers in the *K*. *pneumoniae* strain KP_1.6366. Left panel: fully matched spacer; right panel: 18 and 19 mismatched spacer. The data underlying this figure can be found in S1 Data. PAM, protospacer adjacent motif; SpCas9, *Streptococcus pyogenes* Cas9; WT, wild-type.

xCas9 was reported to recognize diverse PAM sequences, including 5ʹ-NG-3ʹ, 5ʹ-GAA-3ʹ, and 5ʹ-GAT-3ʹ [47]. Given that the NG PAM is the most abundant PAM sequence in most organisms, we sought to assess the activity of xCas9 toward different NG PAM sequences to comprehensively understand the catalytic mechanisms. We used an in vitro DNA cleavage assay to determine the xCas9 activity using linearized plasmids containing various NG PAM sequences as the substrates. As shown in Fig 1A and S1 Fig, the cleavage activity of xCas9 towards plasmids containing TGG, TGC, TGA, AGA, or CGC PAMs was slightly higher than or comparable with that of WT SpCas9. Notably, xCas9 showed significantly higher cleavage activity toward plasmids containing NGT (TGT, CGT, and AGT) PAMs than WT SpCas9.

Next, to examine the DNA-targeting activity of xCas9 in vivo, we substituted the WT SpCas9 gene with the xCas9 gene in the bacterial cytosine base-editing system pBECKP [49], which was constructed by fusing a cytosine deaminase (rat APOBEC1) with a Cas9 nickase (Cas9D10A). Three spacers targeting different genomic loci with TGG, GGT, and GGA PAMs were cloned into both the pBECKP and pBECKP-xCas9 plasmids. The editing efficiency of the resultant spacer-introduced plasmids was assessed using Sanger sequencing, followed by analysis with the EditR software [50]. As shown in Fig 1B, both the pBECKP and pBECKP-xCas9 plasmids could efficiently edit TGG sites, with an average efficiency of 87.9% ± 3.4% for xCas9 and 85.6% ± 2.4% for WT SpCas9. For the CGT and GGT sites, pBECKP-xCas9 showed a much higher editing efficiency (71.5% ± 1.7% and 42.4% ± 1.1% for CGT PAM; 47.9% ± 3.6% for GGT PAM) than WT SpCas9 (2.77% ± 0.17% and 0.57% ± 0.51% for CGT PAM; 6.32% ± 0.46% for GGT PAM), which was consistent with the results in vitro.

In addition to PAM expansion, xCas9 exhibited higher DNA targeting specificity compared with WT SpCas9 [47]. We sought to assess the targeting specificity of xCas9 in bacteria using the pBECKP-xCas9 cytosine base-editing system. We tested five different DNA targets containing NGG PAMs. Both the pBECKP and pBECKP-xCas9 systems showed similar editing efficiencies when the sgRNAs fully matched the DNA targets (Fig 1C, left). However, when two mismatches were introduced into positions 18 and 19 of the spacer sequences, the editing efficiencies of pBECKP-xCas9 were significantly lower than those of pBECKP (Fig 1C, right), demonstrating that xCas9 had higher fidelity in bacteria than WT SpCas9.

### Overall structures of xCas9

To elucidate the molecular mechanisms of the PAM expansion and fidelity enhancement of xCas9, we sought to determine the structures of xCas9 in complex with different PAM sequences. After screening various DNA substrates, we successfully determined the structures of xCas9 in complex with an 83-nucleotide (nt) sgRNA, a 28-nt target DNA strand, and a 12-nt nontarget strand containing different PAM sequences, TGG, CGG, TGA and TGC, at resolutions of 2.90 Å, 2.70 Å, 3.20 Å, and 3.01 Å, respectively (S1 Table).

**Fig 2:**
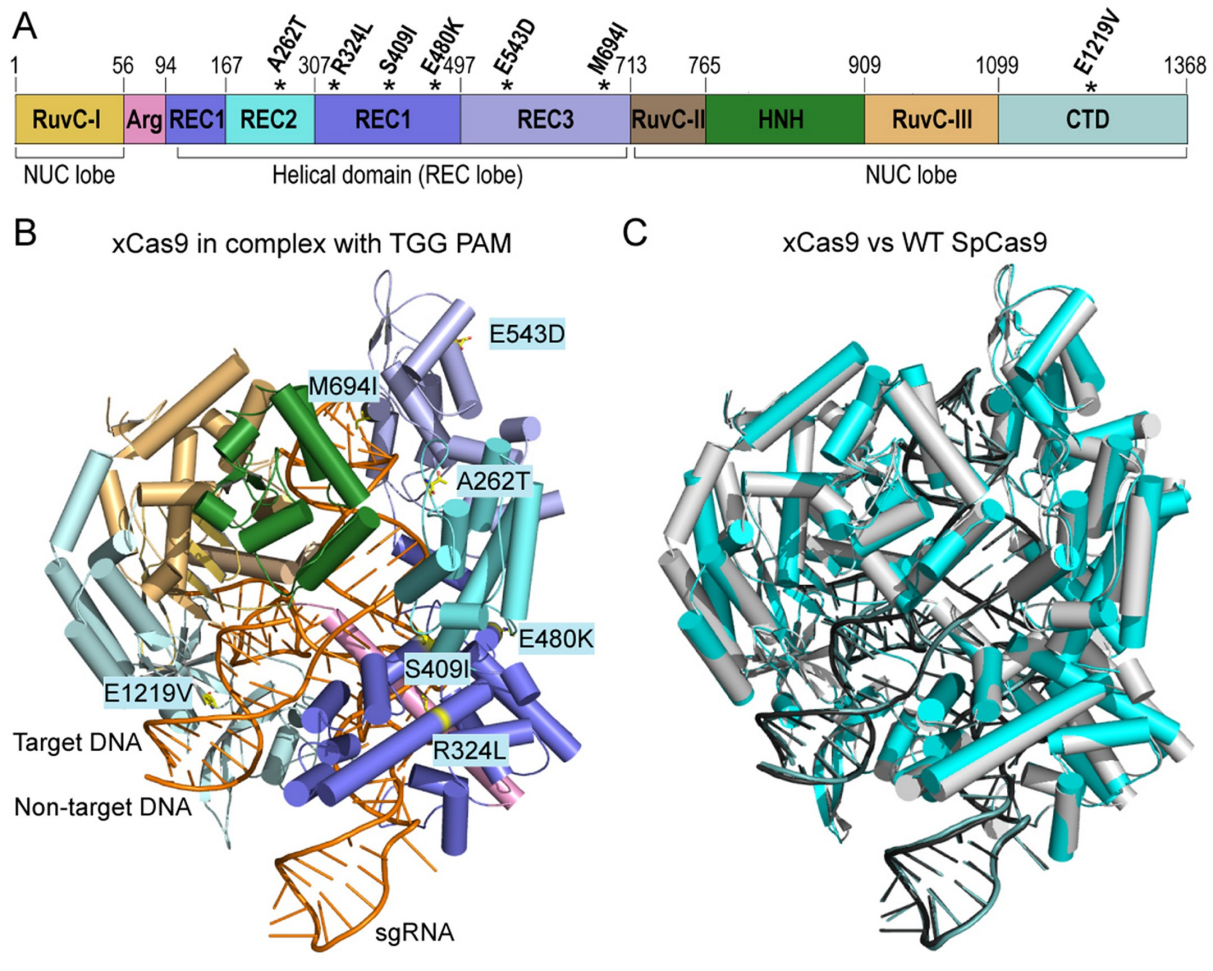
Overall structures of xCas9. (A) Domain organization of xCas9. The positions of seven amino acid substitutions are labeled with asterisk. (B) Cartoon representation showing the overall structure of the xCas9/sgRNA/DNA complex (TGG PAM). (C) Structural comparison of xCas9 (TGG PAM) and WT SpCas9 (PDB_ID: 4UN3). xCas9 and WT SpCas9 are colored cyan and white gray, respectively. CTD, C-terminal domain; HNH, HNH-like nuclease; NUC lobe, nuclease lobe; PAM, protospacer adjacent motif; REC lobe, α-helical recognition lobe; RuvC, RuvC-like nuclease; sgRNA, single-guide RNA; SpCas9, *Streptococcus pyogenes* Cas9; WT, wild-type.

The overall conformations of xCas9 in complex with TGG and CGG PAMs were virtually identical to those of WT SpCas9 (PDB_ID: 4UN3) [10], with a root-mean-square deviation (RMSD) of 0.554 Å and 0.491 Å, respectively (Fig 2A–2C and S2 Fig). Six of the seven mutated residues are located in the REC lobe, including A262T in the REC1 domain; R324L, S409I, and E480K in the REC2 domain; as well as E543D and M694I in the REC3 domain; additionally, the mutation E1219V is located in the CTD domain (Fig 2A). In the SpCas9/sgRNA/DNA complex structure [10], A262, E480, and E543 are solvent-exposed and have no interactions with other residues or nucleotides, suggesting that these three mutations may not contribute to PAM expansion or fidelity enhancement (S3A Fig). M694 forms van der Waals contacts with the last two nucleotides of the RNA/DNA heteroduplex; R324 and S409 are located at the REC1-REC2 interface and are involved in intraresidue interactions (S3A Fig). Given that the reduction in the interactions between the REC domain and DNA/RNA can enhance the fidelity of SpCas9 [44–46], the M694I, R324L, and S409I mutations may contribute to fidelity enhancement by reducing the protein-DNA/RNA interactions in xCas9. E1219 forms a salt bridge with the key PAM recognition residue R1335 (S3B Fig). According to the previous studies [41,48], the mutation of E1219 to V in xCas9 may increase the flexibility of R1335 and thereby contributes to PAM expansion in xCas9.

**Fig 3:**
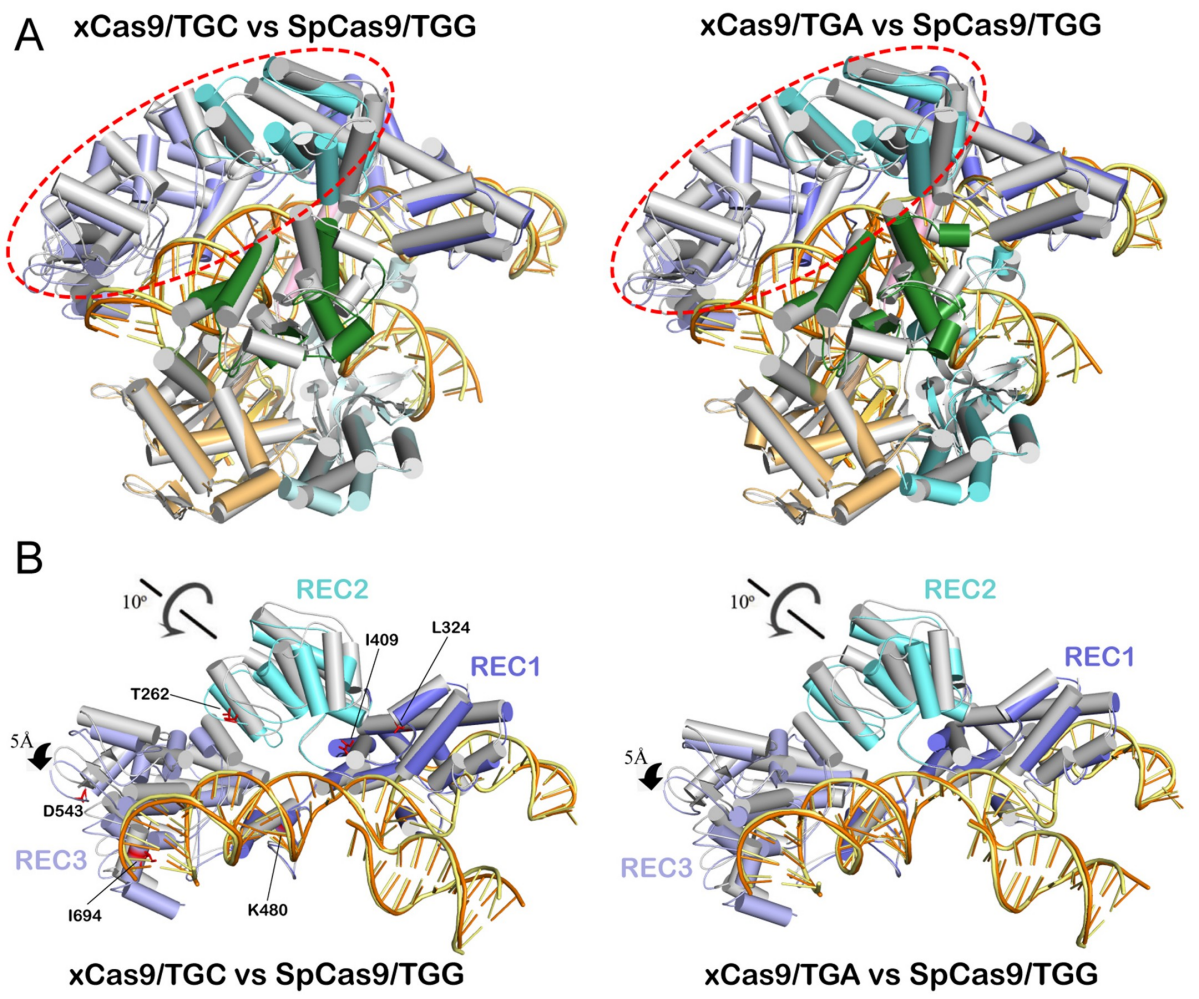
Structural comparison of xCas9 with WT SpCas9. (A) Overall structure superimposition of xCas9 and WT SpCas9 (PDB_ID: 4UN3). WT SpCas9 is colored white gray. Left panel: xCas9/TGC versus WT SpCas9; right panel: xCas9/TGA versus WT SpCas9. (B) Superimposition of the RNA/DNA heteroduplex-bound REC lobe of WT SpCas9 with that of xCas9 in complex with TGC (left) and TGA (right) PAMs. PAM, protospacer adjacent motif; REC lobe, α-helical recognition lobe; SpCas9, *Streptococcus pyogenes* Cas9; WT, wild-type.

The structures of xCas9 in complex with non-NGG PAMs (TGC and TGA PAMs) display significant conformational differences in the REC2 and REC3 domains compared with those in WT SpCas9 (Fig 3A). The REC2 domain in xCas9 rotates approximately 10°, and the REC3 domain moves approximately 5 Å, compared with those in WT SpCas9 (Fig 3B). Despite the remarkable conformational change in the REC domains, the RNA/DNA heteroduplex and other domains of xCas9 maintain a highly conserved conformation. Together, these results reveal that xCas9 employs distinct mechanisms for RNA/DNA recognition when it binds to canonical NGG PAMs and non-NGG PAMs.

### Distinct PAM recognition mechanisms of xCas9 for different PAM sequences

**Fig 4:**
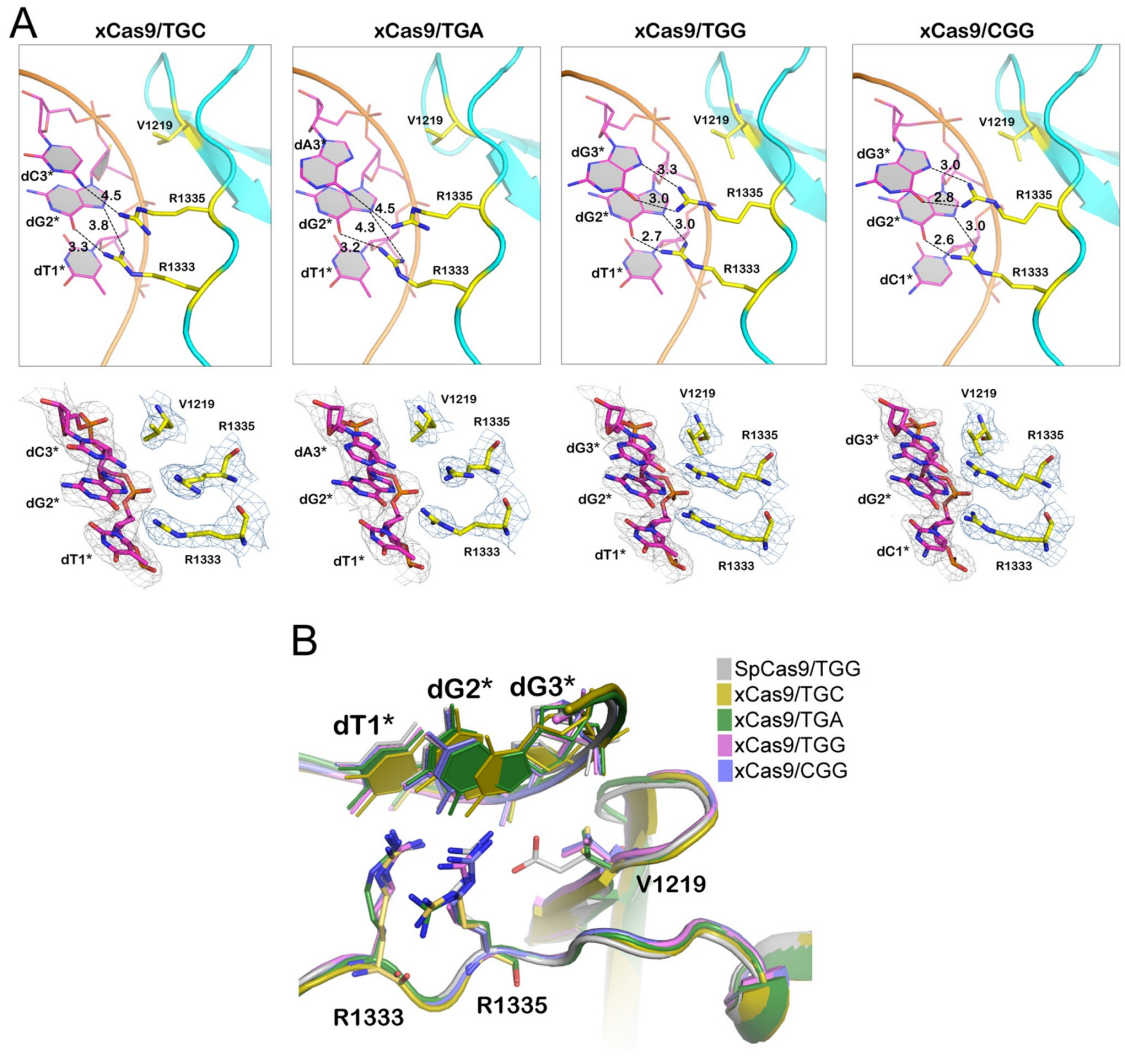
Structural insight into PAM recognition by xCas9. (A) Zoom-in views of PAM recognition by xCas9 in complex of TGC, TGA, TGG, and CGG PAMs, respectively. Upper panel: cartoon-view of PAM recognition in xCas9. Lower panel: The 2F_o_-F_c_ electron density maps for the PAM nucleotides and V1219, R1333, and R1335 residues are contoured at 1.0 sigma level. (B) Superimposition of PAM recognition sites of the TGG PAM-bound SpCas9 with those of TGC, TGA, TGG, and CGG PAM-bound xCas9. PAM, protospacer adjacent motif; SpCas9, *Streptococcus pyogenes* Cas9.

Close inspection of the PAM recognition sites reveals that the E1219V mutation, the only mutation in the CTD domain among the seven mutations, may play an important role in the PAM expansion of xCas9. WT SpCas9 recognizes the NGG PAM through Watson-Crick-like hydrogen bonds between the side chains of R1333 and R1335 and the nucleobases of dG2* and dG3* of the NGG PAM [10]. E1219 forms bidentate salt bridges with R1335 and maintains its stability (S3B Fig). In xCas9, E1219 was substituted by V1219 in xCas9, disrupting the salt bridge interactions with R1335 and rendering R1335 more flexible, consistent with a previous study [48]. In the structures of xCas9 in complex with TGC and TGA PAMs, the side chain of R1335 rotated and lost its direct interaction with dC3* or dA3* (Fig 4A and 4B). In addition, the interaction between R1333 and dG2* was also weakened in the structures of xCas9 in complex with TGC and TGA PAMs. In contrast to the tight bidentate hydrogen bonds (<3.5 Å) between R1333 and dG2* in the structure of WT SpCas9 in complex with the NGG PAM, one of the hydrogen bonds between R1333 and dG2* in the structures of xCas9 in complex with TGC and TGA PAMs was increased to 3.8 Å and 4.3 Å, respectively (Fig 4A). Intriguingly, in the structures of xCas9 in complex with canonical PAMs (TGG and CGG), the conformations of R1333 and R1335 are similar to those observed in the structure of WT SpCas9 in complex with NGG PAMs (Fig 4A and 4B), demonstrating that xCas9 utilizes distinct modes for the recognition of different PAMs.

### Crystal structures of SpCas9 in complex with non-NGG PAMs

**Fig 5:**
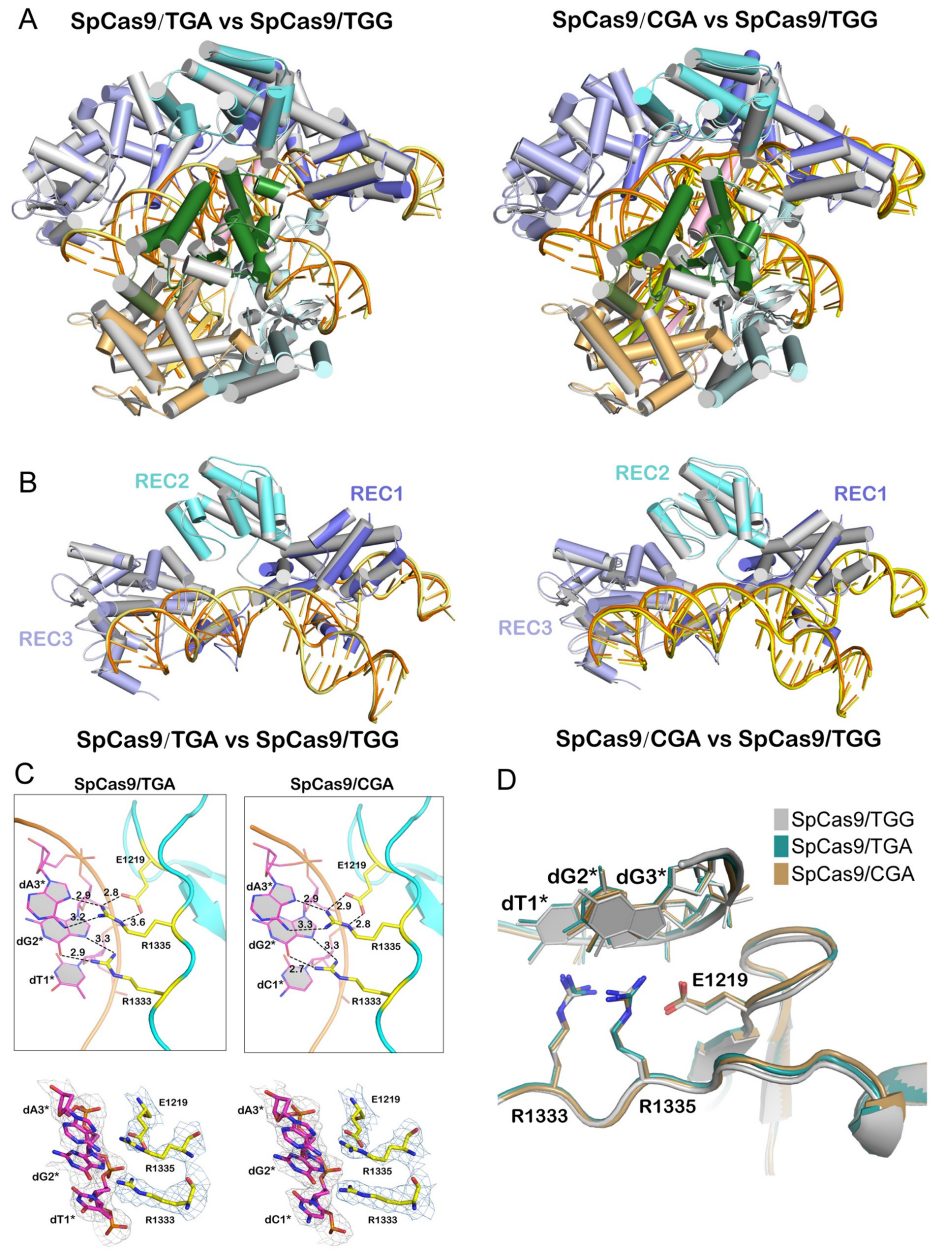
Structural comparison of SpCas9/TGA and SpCas9/CGA with SpCas9/TGG. (A) Superimposition of the overall structures of SpCas9/TGA and SpCas9/CGA with SpCas9/TGG (PDB_ID: 4UN3). (B) Superimposition of the REC lobe of SpCas9/TGG with those of SpCas9 in complex with TGA (left) and CGA (right) PAMs. (C) Close-up view of the PAM recognition sites of TGA and CGA PAM-bound SpCas9. Upper panel: cartoon-view of PAM recognition in SpCas9. Lower panel: The 2F_o_-F_c_ electron density maps are contoured at 1.0 sigma level for the PAM nucleotides and V1219, R1333, and R1335. (D) Structural superimposition of PAM recognition sites of the TGG PAM-bound SpCas9 with those of TGA and CGA PAM-bound SpCas9. In (A) and (B), color codes of SpCas9/TGA and SpCas9/CGA are the same as those shown in Fig 1A, and SpCas9/TGG is colored in white gray. PAM, protospacer adjacent motif; REC lobe, α-helical recognition lobe; SpCas9, *Streptococcus pyogenes* Cas9; WT, wild-type.

The structural characterizations of xCas9 in complex with canonical NGG PAMs and non-NGG PAMs revealed that xCas9 employs distinct mechanisms for DNA/RNA binding and PAM recognition when it binds to different PAM sequences. To examine whether these mechanisms are unique for xCas9, we further determined the structures of WT SpCas9 in complex with two noncanonical PAMs (TGA and CGA; S1 Table). The structures of SpCas9/TGA and SpCas9/TGA were almost identical to the structure of SpCas9 in complex with the TGG PAM (PDB_ID: 4UN3), with RMSD values of 0.282 Å and 0.337 Å, respectively (Fig 5A and 5B). In addition, R1333 and R1335 in TGA and CGA PAM-bound SpCas9 exhibited similar conformations as those in TGG PAM-bound SpCas9 (Fig 5C and 5D). These results demonstrate that WT SpCas9 retains a highly conserved conformation even when it binds to non-NGG PAMs, and the distinct mechanisms for DNA/RNA binding and PAM recognition are unique for xCas9.

### Examining the key residues for PAM expansion and fidelity

**Fig 6:**
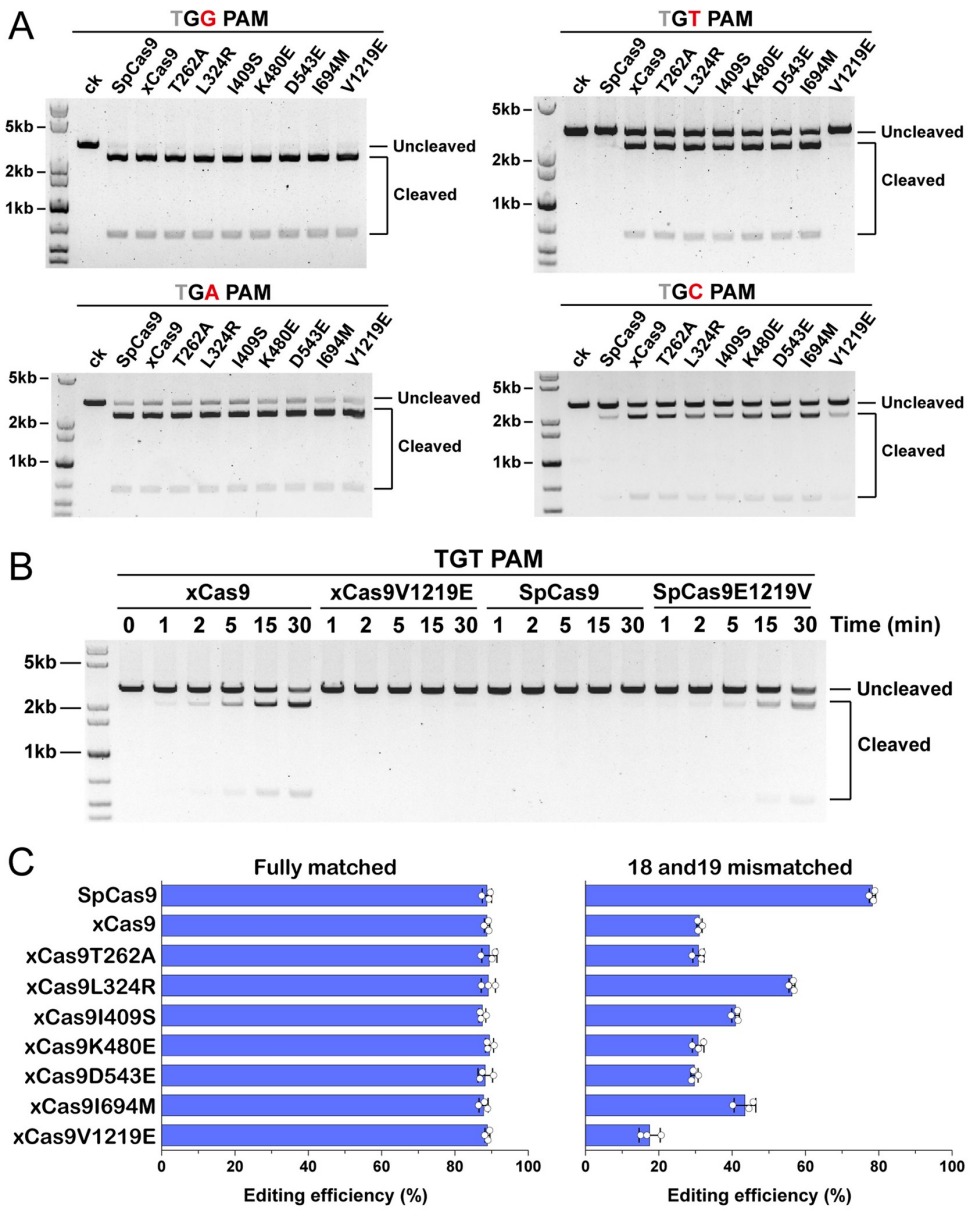
In vitro and in vivo activity of WT SpCas9, xCas9, and xCas9 variants. (A) In vitro cleavage assays of WT SpCas9, xCas9, and xCas9 variants using linearized plasmids containing a target sequence adjacent to TGG, TGA, TGT, and TGC PAMs. (B) In vitro cleavage assays of xCas9, xCas9V1219E, WT SpCas9, and SpCas9E1219V toward TGT targets. (C) Cytosine base editing efficiency of pBECKP, pBECKP-xCas9, and pBECKP-xCas9 variant plasmids in the *K*. *pneumoniae* strain KP_1.6366. Left panel: fully matched spacer; right panel: 18 and 19 mismatched spacer. The data underlying this figure can be found in S1 Data. PAM, protospacer adjacent motif; SpCas9, *Streptococcus pyogenes* Cas9; WT, wild-type.

To systematically study the contributions of each mutation to PAM expansion and fidelity enhancement, we individually mutated the seven amino acid substitutions in xCas9 back to the WT SpCas9 residues. Seven xCas9 variant proteins (T262A, L324R, I409S, K480E, D543E, I694M, and V1219E) were purified (S4 Fig) and subjected to the in vitro DNA cleavage assay. As shown in Fig 6A, the mutation of V1219 back to E significantly reduced the cleavage activity of xCas9 towards DNAs containing TGT or TGA PAMs, whereas no significant activity differences were observed in the other six xCas9 variants compared with that in xCas9. In addition, we introduced a single mutation, E1219V, in WT SpCas9 and tested its DNA cleavage activity towards the TGT PAM. As shown in Fig 6B, the mutant protein SpCas9E1219V exhibited significantly enhanced cleavage activity for the TGT PAM-containing substrate compared to that in WT SpCas9 (Fig 6B), thus confirming the key role of the E1219V mutation in PAM expansion.

We further sought to identify the key residues responsible for the observed enhancement of the DNA-targeting specificity of xCas9. Seven single amino acid substitutions were introduced into the pBECKP-xCas9 plasmid. All mutated plasmids could perform effective cytosine base editing against the fully matched spacer with almost the same efficiencies (Fig 6C, left). Then, we introduced two mismatches at positions 18 and 19 of the spacer and assessed the cytosine base-editing efficiencies of the seven pBECKP-xCas9 variant plasmids again. Consistent with the observations from the crystal structures, the single mutation of the three solvent-exposed residues (T262A, K480E, and D543E) in xCas9 had little effect on the base-editing activity towards mismatched DNA substrates (Fig 6C, right). Remarkably, the editing efficiency was significantly increased when the substitution of L324R, I409S, or I694M was introduced into pBECKP-xCas9, suggesting that these three residues play an important role in fidelity enhancement (Fig 6C, right). These results are consistent with the notion that mutations in the high-fidelity hotspot interfaces of the REC domain can improve the DNA targeting specificity of the SpCas9 nuclease [44–46,48]. Intriguingly, the mutation of V1219 back to E in xCas9 decreased the off-target editing activity (Fig 6C, right), suggesting that interference in the CTD domain may also affect the DNA-targeting specificity of xCas9.

## Discussion

In this study, we systematically investigated the genome editing capacity of xCas9 using both in vitro cleavage assays and in vivo bacterial cell-based genome editing experiments. Moreover, we determined the crystal structure of xCas9 in complex with sgRNA and double-stranded DNA containing TGG, CGG, TGA, and TGC PAM sequences. Structural analysis revealed that xCas9 undergoes a notable conformational rearrangement in the REC2 and REC3 domains. In addition, the key PAM-interacting residue R1335 rotates when it recognizes non-NGG DNA substrates. In contrast, the structures of xCas9 in complex with canonical NGG PAMs are virtually identical to those of WT SpCas9. This two-mode PAM recognition mechanism is unique for xCas9 because WT SpCas9 adopts similar conformation in non-NGG and TGG PAM recognition. In addition, in vitro and in vivo assays demonstrated that the E1219V mutation is critical for lessening the stringent requirements for the PAM sequence in xCas9, and the R324L, S409I, and M694I mutations in the REC lobe are responsible for the high DNA-targeting specificity of xCas9. The data presented here advance our mechanistic understanding of the broadened PAM compatibility and enhanced fidelity of xCas9.

Although the crystal structure of xCas9 in complex with GAT or GAA PAMs has been reported recently [48], our structures revealed new notable features of xCas9. In this work, we discovered that xCas9 in complex with canonical NGG PAMs adopted the same configuration as WT SpCas9. The structural rearrangement in the REC lobe and the rotation of the PAM-interacting residues only occurred in the xCas9 structures complexed with non-NGG PAMs. Notably, the rotation amplitude of the REC domain in xCas9 in complex with TGC and TGA PAMs was significantly different from that in the structures of xCas9/GAT and xCas9/AAG (S5 Fig) [48]. In addition, in the structures of the xCas9/GAT and xCas9/GAA complexes, R1335 stayed in a similar conformation as that in the structure of WT SpCas9, whereas R1333 rotated flexibly [48]. Accordingly, in our structures of TGC and TGA PAM-bound xCas9, R1333 remains in the same position as that in the structure of WT SpCas9, whereas R1335 rotates in another direction, resulting in the disruption of the interaction between R1335 and the third nucleotide of the PAM sequence (S6 Fig). These results revealed that xCas9 adopted distinct structural conformations to recognize different PAM sequences, which is likely attributable to the flexibility caused by the seven amino acid substitutions.

The REC lobe of the Cas9 nuclease recognizes the guide RNA scaffold and RNA/DNA heteroduplex and plays a crucial role in DNA cleavage and off-target discrimination [10,45,51,52]. The conformational rearrangement in the REC lobe is unique to xCas9 and is not observed in the other reported structures of PAM-expanded SpCas9 variants [40–42]. A previous study speculated that these conformational changes impair the interaction between the REC lobe and DNA substrates, thus contributing to the enhancement of the DNA-targeting specificity of xCas9 [48]. However, our work reveals that xCas9 remains in the same conformation as WT SpCas9 when it recognizes canonical NGG PAMs, even though the DNA fidelity of xCas9 towards NGG targets was improved. This result implies that the enhanced DNA specificity of xCas9 may not result from the REC lobe conformational rearrangement but is likely the result of the mutations of residues that weaken the interactions between SpCas9 and the DNA substrate.

## Materials and methods

### xCas9 expression and purification

The full-length xCas9 gene was synthesized and subcloned into a pET28a-modified vector that expresses the fusion protein containing an N-terminal His_6_-tag followed by a human rhinovirus 3C (HRV3C) protease cleavage site. For crystallization, D10 and H840 in xCas9 were mutated to Ala by using the site-directed mutagenesis. Because the mutations of C80 and C574 to Leu and Glu in the SpCas9 protein improved the crystal quality [19], C80 and C574 in xCas9 were also mutated to Leu and Glu, respectively. The constructed plasmid was transformed into *Escherichia coli* BL21 (DE3) for protein expression. The cells were cultured in the LB medium containing 50 μg/mL kanamycin at 37°C, and the expression of xCas9 was induced by the addition of 0.5 mM IPTG when OD_600_ of the cells reached to 0.6. The *E*. *coli* cells were further incubated at 16°C overnight before being harvested.

Cells were lysed in 30 mL buffer A (10 mM Tris-HCl [pH 7.4], 500 mM NaCl, 5% glycerol, 1 mM DTT), and then centrifuged at 14,000*g* for 30 min. The supernatant was loaded onto a Ni-NTA column (GE Healthcare, Beijing, China) and the His-tagged protein was eluted with buffer B (10 mM Tris-HCl [pH 7.4], 500 mM NaCl, 500 mM imidazole, 5% glycerol, 1 mM DTT). After digestion with the HRV3C protease overnight, the protein solution was clarified and passed through a Ni-NTA column to remove uncleaved xCas9 and the HRV3C protease. The cleaved xCas9 protein was concentrated to 2 mL using an Amicon Ultra 10K filter (Millipore, Burlington, MA), and was further purified by a size-exclusion chromatography using a HiLoad Superdex 200 16/60 column (GE Healthcare, Beijing, China).

xCas9 variants were constructed by introducing point mutations into xCas9 using site-directed mutagenesis and verified by DNA sequencing. The purification processes of SpCas9 and xCas9 variant proteins were the same as that of xCas9.

### Preparation of sgRNA and DNA

The transcription template (double-stranded DNA) for sgRNA was synthesized as a gblock (S2 Table) and was amplified by PCR. sgRNA was transcribed in vitro using the HiScribe T7 High Yield RNA Synthesis Kit (New England Biolabs, Ipswich, MA). After incubated at 37°C for 16 h, the reaction solution was treated with DNase I (RNase free) to eliminate the DNA template. sgRNA was purified using phenol/chloroform extraction followed by ethanol precipitation. Finally, sgRNA was dissolved in DEPC water and stored at −80°C.

The target and non-target DNA strands for crystallization were purchased from Sangon (Shanghai, China; S2 Table). Two oligos were dissolved in a solution containing 10 mM Tris-HCl (pH 7.4) and 50 mM NaCl and were mixed with a 1:1 molar ratio. The mixture was heated at 85°C for 5 min and then cooled down slowly to room temperature. The annealed double-stranded DNA was stored at −80°C.

### Crystallization and structure determination

The purified xCas9 protein was mixed with sgRNA and DNA (molar ratio, 1:1.3:1.7) and incubated on ice for 30 min. The xCas9/sgRNA/DNA complex was purified in a buffer containing 10 mM Tris-HCl (pH 7.4), 150 mM NaCl, 5 mM MgCl_2_, and 1mM DTT by size-exclusion chromatography using a Superdex200 16/600 column.

The purified complex was concentrated to 10 mg/mL and crystallized at 16°C by using the hanging-drop vapor diffusion method. Crystals were obtained by mixing 1 μl of the complex solution and 1 μl of the reservoir solution (0.2 M Ammonium phosphate dibasic, 20% w/v Polyethylene glycol 3,350 [pH 8.0]). The crystals were cryo-protected and flash-frozen in liquid nitrogen for X-ray diffraction.

X-ray diffraction data was collected at BL19U1 beamline of National Facility for Protein Science Shanghai (NFPS) at Shanghai Synchrotron Radiation Facility. HKL3000 program was used to integrate and scale the X-ray data. Structures of xCas9 in complex with sgRNA and DNA were resolved by molecular replacement with PHASER from CCP4i using the structure of SpCas9 (PDB_ID: 4UN3) [10] as the searching model. The structure models were improved manually using COOT and refined using Refmac5 from CCP4i. Structural figures were prepared using Pymol (http://www.pymol.org).

### DNA cleavage assay

The DNA cleavage assay was performed using the methods described previously by Anders and colleagues and Nishimasu and colleagues [41,42]. Briefly, all target plasmids were constructed by inserting a DNA sequence comprising a 20-nt target site and an adjacent PAM into the *BamH*I/*EcoR*I sites of the pUC19 plasmid. The target plasmids were linearized by *Ssp*I and the linearized products were used as the DNA substrates for the cleavage assay. A total of 200 nM xCas9 and 400 nM sgRNA were incubated in the CutSmart Buffer (New England Biolabs, Ipswich, MA) at room temperature for 5 min. Next, 5 nM (final concentration) linearized target plasmid was added into the mixture, and the reaction was further incubated at 37°C for 30 min. Aliquots (10 μL) were taken at indicated time points, and the reactions were stopped by the addition of 25 mM EDTA. After treated with 10 μg Proteinase K at room temperature for 30 min, the cleavage products were analyzed by a 1% agarose gel. The separated products were stained with the 4S red plus dye (Sangon, Shanghai, China) and visualized by the ChemiDoc MP System (Bio-Rad, Hercules, CA).

### Cytosine base editing assay in *K*. *pneumoniae*

The xCas9 gene and the backbone of pBECKP [49] were PCR-amplified, respectively. They were assembled together via Gibson assembly [53] to form the plasmid pBECKP-xCas9. The success of the construction of pBECKP-xCas9 plasmid was verified by PCR, enzyme digestion, and sequencing. Point mutations were introduced into xCas9 using site-directed mutagenesis and confirmed by DNA sequencing.

The cytosine base editing plasmids were constructed by inserting spacers into the *Bsa*I sites of the pBECKP and pBECKP-xCas9 plasmids using Golden Gate assembly [54]. The constructed editing plasmids were electroporated into the *K*. *pneumoniae* KP_1.6366 strain with the parameters of 1800 V, 200 Ω, and 25 μF (1 mm cuvette). The cells were plated onto an LB agar plate containing 50 μg/mL kanamycin. After incubation overnight at 30°C, all the colonies were collected from the plate and applied for genome extraction using the Ezup column bacteria genomic DNA purification kit (Sangon, Shanghai, China). PCR products covering the editing sites were sent out for Sanger sequencing, and the editing efficiency was quantified using EditR software [50].

## Supporting information

S1 Fig In vitro cleavage assays of xCas9 and WT SpCas9 using linearized plasmids containing a target sequence adjacent to CGT, AGT, AGA, and CGC PAMs. PAM, protospacer adjacent motif; SpCas9, *Streptococcus pyogenes* Cas9; WT, wild-type.
(TIF)

S2 Fig Structural superimposition of xCas9 (CGG PAM) and WT SpCas9 (TGG PAM, PDB_ID: 4UN3). xCas9 is colored orange and WT SpCas9 is colored white gray. PAM, protospacer adjacent motif; SpCas9, *Streptococcus pyogenes* Cas9; WT, wild-type.
(TIF)

S3 Fig Positions of seven mutated amino acids in SpCas9 (PDB_ID: 4UN3). (A) Detailed locations of six amino acid substitutions in the REC lobe. (B) Position of E1219 and its interaction with the PAM recognition residue R1335. PAM, protospacer adjacent motif; REC lobe, α-helical recognition lobe; SpCas9, *Streptococcus pyogenes* Cas9.
(TIF)

S4 Fig The SDS-PAGE image of the purified WT SpCas9, SpCas9E1219V, xCas9, and xCas9 variants (T262A, L324R, I409S, K480E, D543E, I694M, and V1219E). SpCas9, *Streptococcus pyogenes* Cas9; WT, wild-type.
(TIF)

S5 Fig Structural comparison of the RNA/DNA heteroduplex-bound REC lobe of xCas9/TGC with that of xCas9/GAT (PDB_ID: 6AEG). REC lobe, α-helical recognition lobe.
(TIF)

S6 Fig Structural superimposition of PAM recognition sites of the TGG PAM-bound SpCas9 with TGC, GAT, and AAG PAM-bound xCas9. PAM, protospacer adjacent motif; SpCas9, *Streptococcus pyogenes* Cas9.
(TIF)

S1 Table Data collection, phasing, and refinement statistics of the structures of xCas9 and SpCas9 in complex with different PAMs. PAM, protospacer adjacent motif; SpCas9, *Streptococcus pyogenes* Cas9.
(DOCX)

S2 Table The sequences of nucleic acids used in this study. (DOCX)

S1 Data Excel file containing the numerical editing efficiency data for Figs 1B, 1C and 6C. (XLSX)

S1 Raw Images Original images of agarose gels shown in Figs 1 and 6 and S1 Fig and the SDS-PAGE in S4 Fig. (PDF)

## Abbreviations

CRISPR: Clustered regularly interspaced short palindromic repeats
crRNA: CRISPR RNA
CTD: C-terminal domain
HNH: HNH-like nuclease
HRV3C: human rhinovirus 3C
NFPS: National Facility for Protein Science Shanghai
nt: nucleotide
NUC lobe: nuclease lobe
PAM: protospacer adjacent motif
REC lobe: α-helical recognition lobe
RMSD: root-mean-square deviation
RuvC: RuvC-like nuclease
sgRNA: single-guide RNA
SpCas9: *Streptococcus pyogenes* Cas9
tracrRNA: transactivating crRNA
WT: wild-type
