# Supplementary figures and images for: Molecular basis for the PAM expansion and fidelity enhancement of an evolved Cas9 nuclease
Source: PLoS Biol. 2019 Oct 11;17(10):e3000496. doi: 10.1371/journal.pbio.3000496 (PMC6808508; doi:10.1371/journal.pbio.3000496)

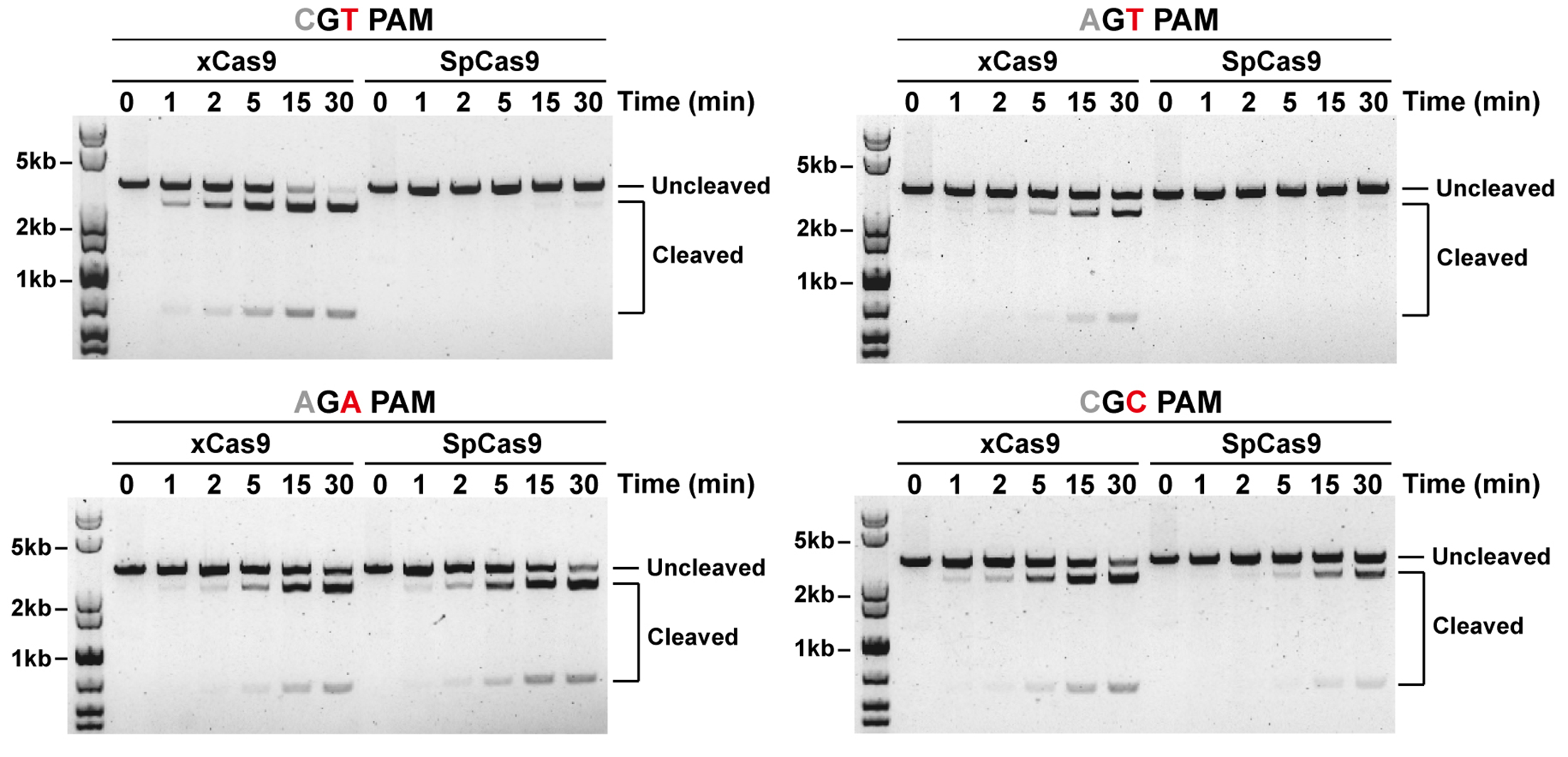

Supplement: S1 Fig — PAM, protospacer adjacent motif; SpCas9, Streptococcus pyogenes Cas9; WT, wild-type. (TIF) [file pbio.3000496.s001.tif]

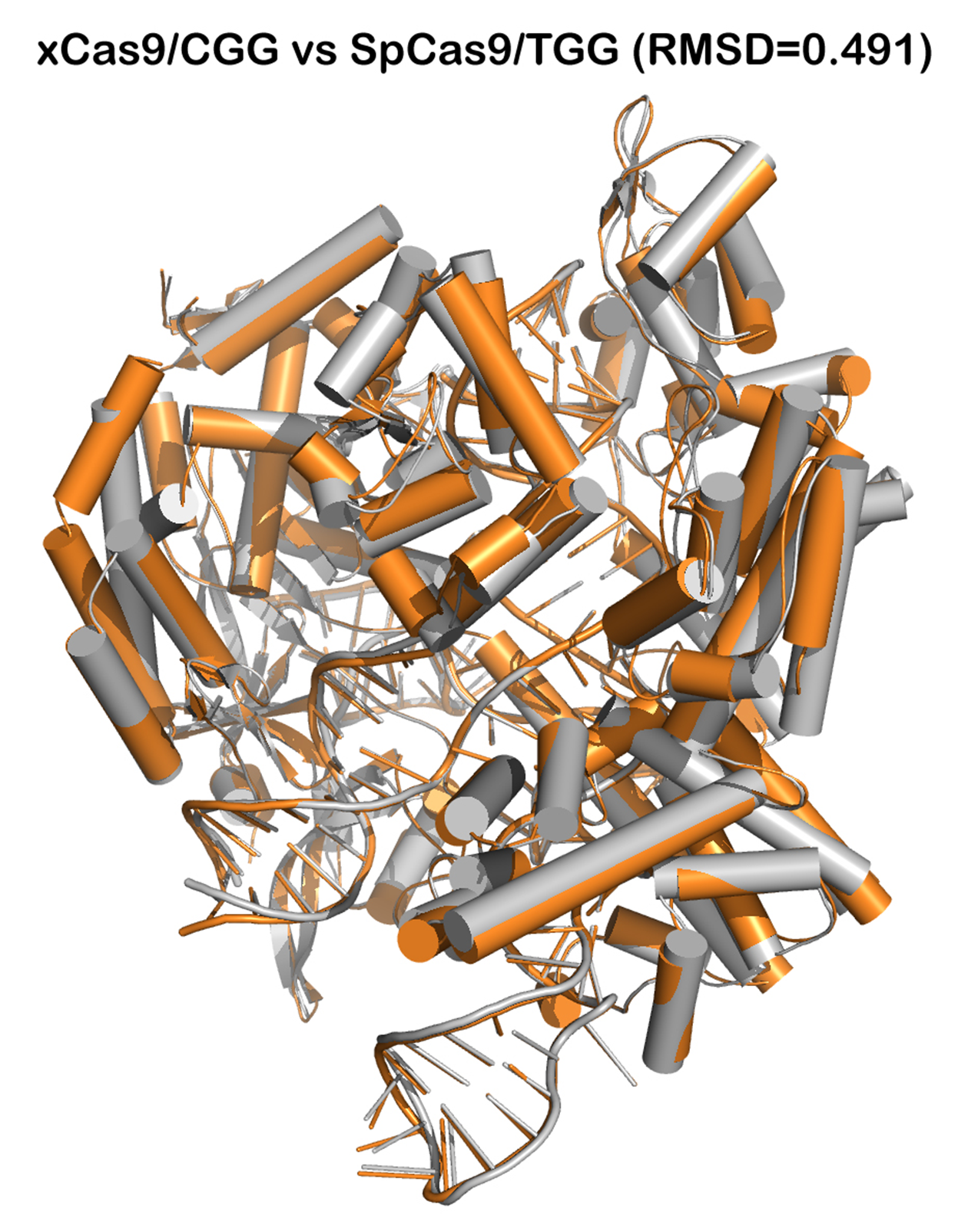

Supplement: S2 Fig — xCas9 is colored orange and WT SpCas9 is colored white gray. PAM, protospacer adjacent motif; SpCas9, Streptococcus pyogenes Cas9; WT, wild-type. (TIF) [file pbio.3000496.s002.tif]

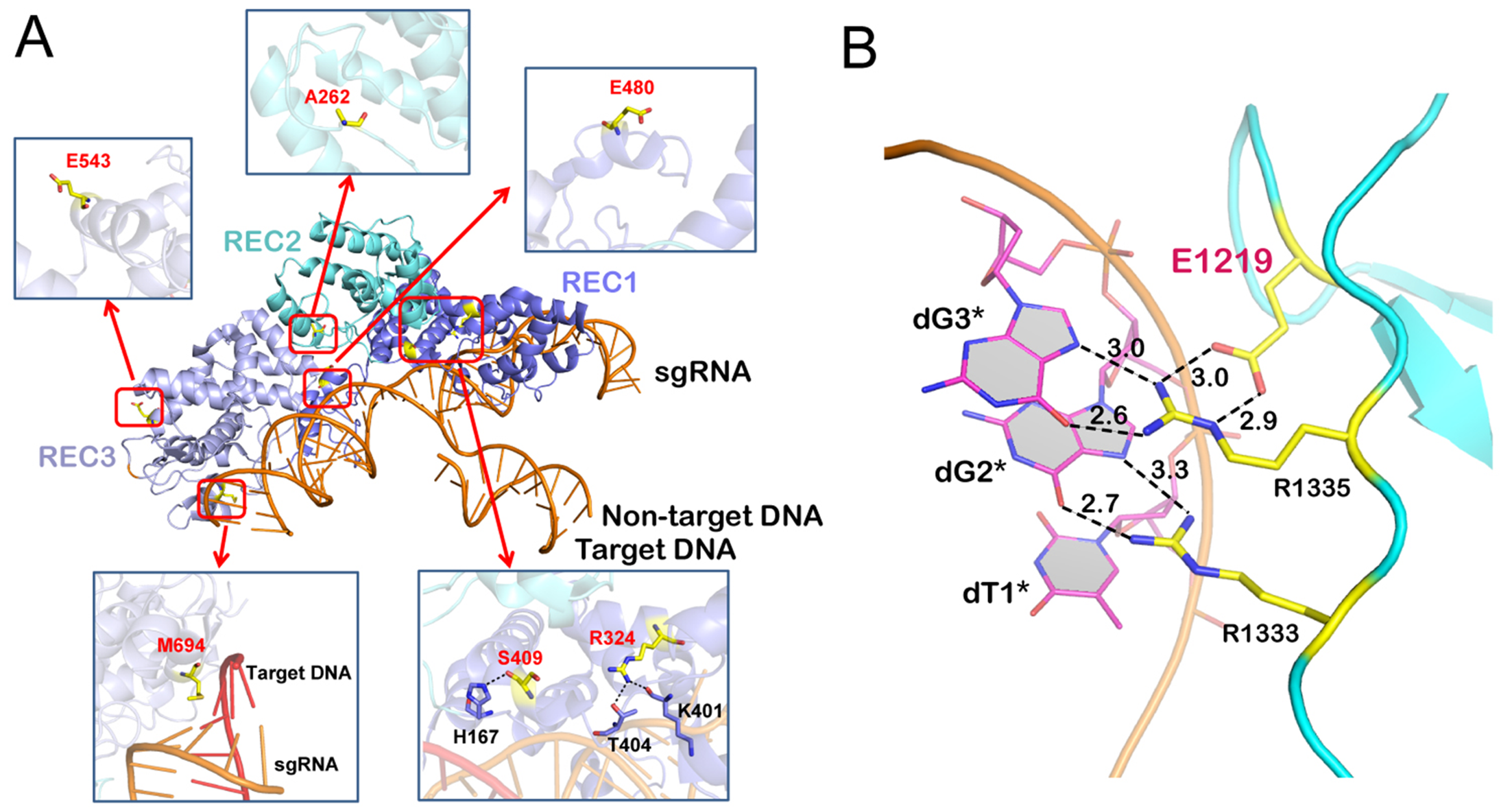

Supplement: S3 Fig — (A) Detailed locations of six amino acid substitutions in the REC lobe. (B) Position of E1219 and its interaction with the PAM recognition residue R1335. PAM, protospacer adjacent motif; REC lobe, α-helical recognition lobe; SpCas9, Streptococcus pyogenes Cas9. (TIF) [file pbio.3000496.s003.tif]

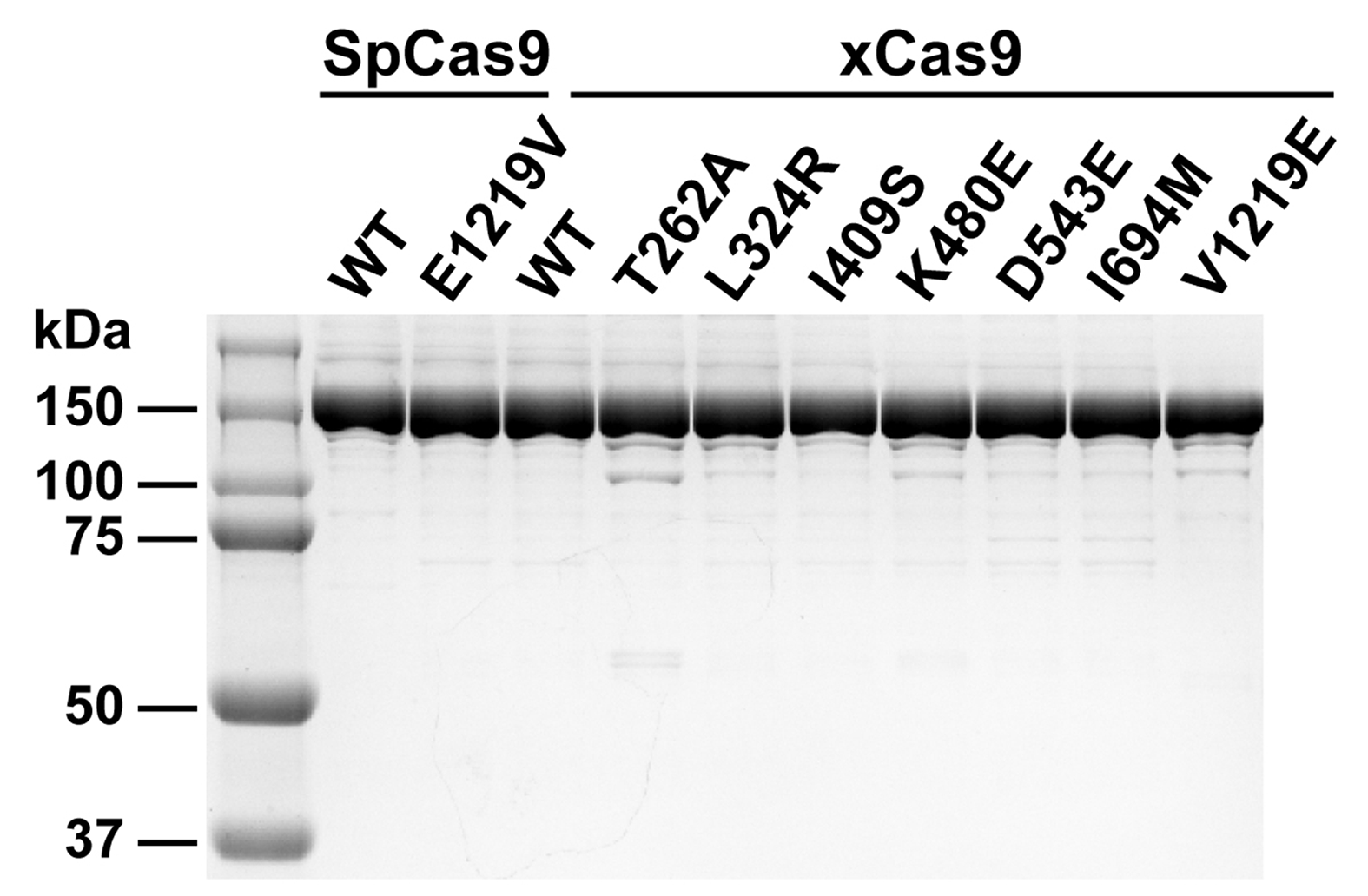

Supplement: S4 Fig — SpCas9, Streptococcus pyogenes Cas9; WT, wild-type. (TIF) [file pbio.3000496.s004.tif]

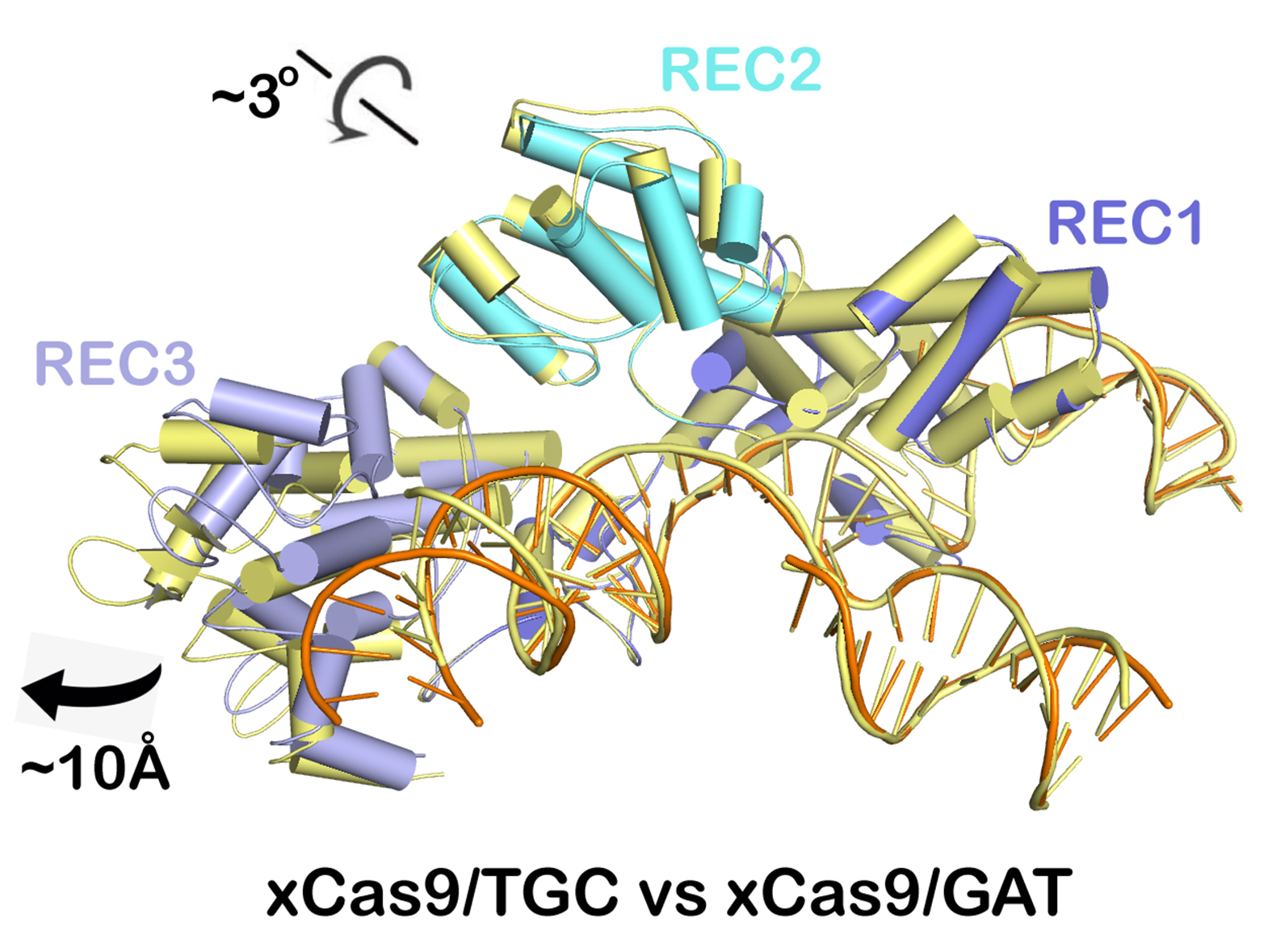

Supplement: S5 Fig — REC lobe, α-helical recognition lobe. (TIF) [file pbio.3000496.s005.tif]

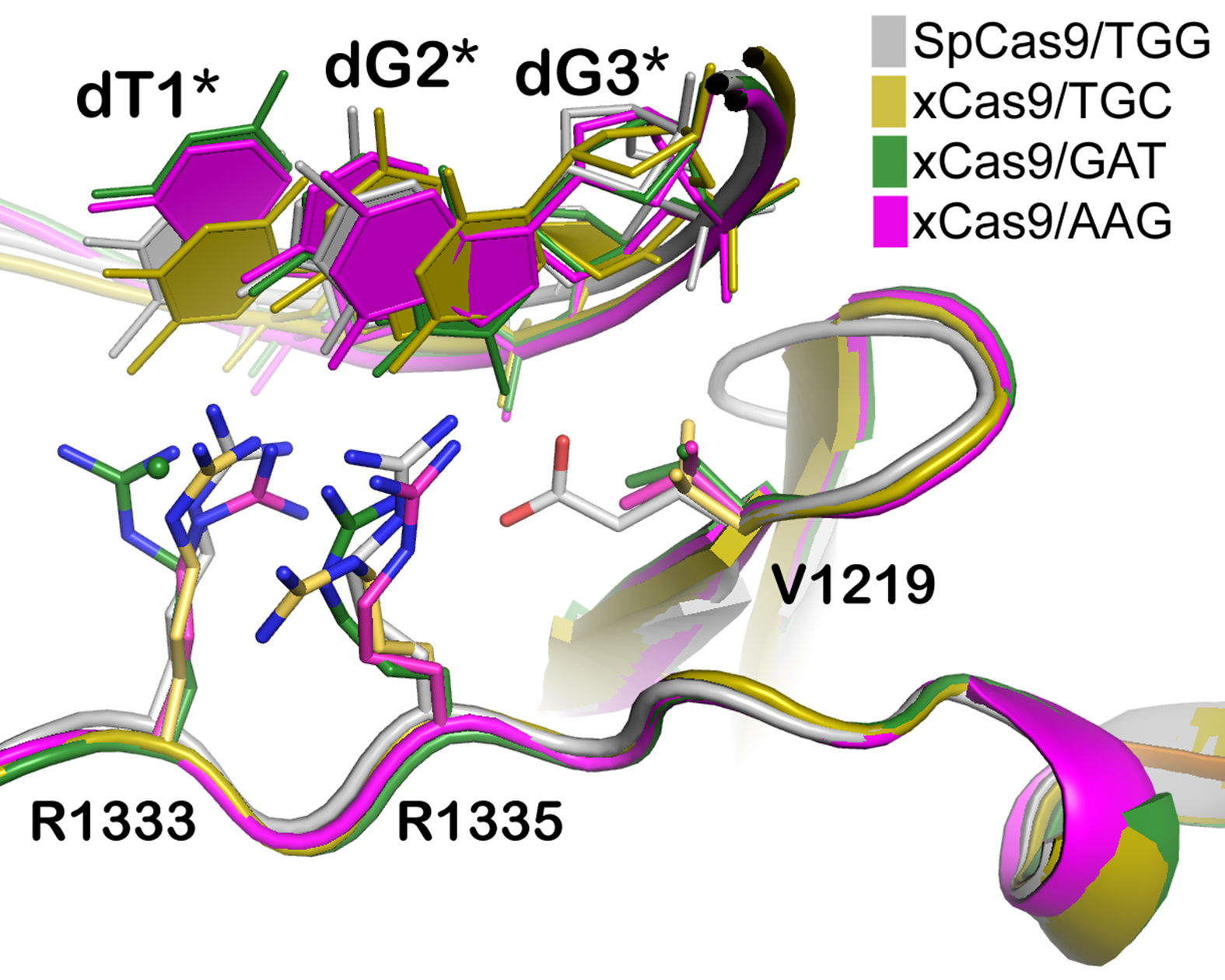

Supplement: S6 Fig — PAM, protospacer adjacent motif; SpCas9, Streptococcus pyogenes Cas9. (TIF) [file pbio.3000496.s006.tif]
