# Supplementary material for: Molecular basis for the PAM expansion and fidelity enhancement of an evolved Cas9 nuclease
Source: PLoS Biol. 2019 Oct 11;17(10):e3000496. doi: 10.1371/journal.pbio.3000496 (PMC6808508; doi:10.1371/journal.pbio.3000496)
Supplement: S1 Table — PAM, protospacer adjacent motif; SpCas9, Streptococcus pyogenes Cas9. (DOCX) [file pbio.3000496.s007.docx]

**S1 Table.** Data collection, phasing, and refinement statistics of the structures of xCas9 and SpCas9 in complex with different PAMs.

|  | xCas9/CGG | xCas9/TGG | xCas9/TGA | xCas9/TGC | SpCas9/CGA | SpCas9/TGA |
| --- | --- | --- | --- | --- | --- | --- |
| Wavelength (Å) | 0.9785 | 0.9785 | 0.9785 | 0.9785 | 0.9785 | 0.9785 |
| Beamline^a^ | BL19U1 | BL19U1 | BL19U1 | BL19U1 | BL19U1 | BL19U1 |
| Space group | C2 | C2 | C2 | C2 | C2 | C2 |
| Cell dimensions |  |  |  |  |  |  |
| a, b, c (Å) | 177.84, 69.669, 189.513 | 177.785, 69.667, 189.016 | 178.005, 70.314, 190.077 | 177.464, 70.364, 188.899 | 177.956, 69.137, 189.243, | 177.943, 69.058, 190.046, |
| α, β, γ (°) | 90, 109.354, 90 | 90, 109.521, 90 | 90, 110.343, 90 | 90, 110.415, 90 | 90, 110.342, 90, | 90, 110.205, 90, |
| Resolution (Å) | 50.00-2.70 | 50.00-2.90 | 50.00-3.20 | 50.00-3.01 | 50.00-2.98 | 50.00-3.20 |
| R_merge_ (%) | 15.2 (111.9)^b^ | 17.0 (99.7) | 16.3 (120.2) | 16.9 (127.9) | 10.9 (86.6) | 14.3 (91.5) |
| I/σI | 11.6 (1.67) | 6.8 (1.00) | 10.2 (1.00) | 12.0 (1.00) | 16.2 (2.00) | 12.1 (1.82) |
| Completeness (%) | 99.8 (99.7) | 98.9 (99.4) | 99.0 (98.9) | 98.8 (96.9) | 98.8 (97.2) | 98.3 (97.7) |
| Redundancy | 6.7 (5.7) | 3.0 (2.9) | 4.8 (4.3) | 6.5 (5.5) | 6.4 (5.7) | 6.4 (6.2) |
| Refinement |  |  |  |  |  |  |
| Resolution (Å) | 29.95-2.70 | 29.85-2.90 | 44.24-3.20 | 44.04-3.01 | 44.36-2.98 | 40.89-3.20 |
| Reflections (#) | 57071 | 44803 | 30154 | 38197 | 41003 | 31672 |
| R_work_/R_free_ (%) | 20.9/26.9 | 20.1/26.8 | 25.1/28.6 | 24.1/28.3 | 20.2/26.6 | 20.7/26.4 |
| Number of nonhydrogen Atoms (#) |  |  |  |  |  |  |
| Protein/NA | 13061 | 13073 | 12818 | 12412 | 13288 | 13288 |
| PO_4_^3-^ | 45 | 25 | 0 | 0 | 0 | 0 |
| H_2_O  Rmsds | 99 | 22 | 0 | 0 | 22 | 0 |
| Bond lengths (Å) | 0.011 | 0.012 | 0.006 | 0.006 | 0.013 | 0.014 |
| Bond angle (°) | 1.47 | 1.65 | 0.77 | 0.81 | 1.97 | 2.08 |
| Average B factors (Å^2^) |  |  |  |  |  |  |
| Overall | 26.72 | 25.52 | 74.28 | 76.61 | 61.99 | 61.53 |
| Protein/NA | 26.57 | 25.38 | 74.28 | 76.61 | 62.03 | 61.53 |

^a^Crystal data were collected at the BL19U1 beamline of National Facility for Protein Science Shanghai (NFPS) at Shanghai Synchrotron Radiation Facility.

^b^Statistics for the highest-resolution shell are shown in parentheses.
