# Supplementary material for: Molecular basis for the PAM expansion and fidelity enhancement of an evolved Cas9 nuclease
Source: PLoS Biol. 2019 Oct 11;17(10):e3000496. doi: 10.1371/journal.pbio.3000496 (PMC6808508; doi:10.1371/journal.pbio.3000496)
Supplement: S2 Table — (DOCX) [file pbio.3000496.s008.docx]

**S2 Table.** Sequences of nucleic acids used in this study

|  | **Name** | **Sequence (5’-3’)** | **Description** |
| --- | --- | --- | --- |
| sgRNA expression | sgRNA | GGAUAACUCAAUUUGUAAAAAAGUUUUAGAGCUAGAAAUAGCAAGUUAAAAUAAGGCUAGUCCGUUAUCAACUUGAAAAAGUG | sequence of sgRNA |
|  | DNA template | CACTTTTTCAAGTTGATAACGGACTAGCCTTATTTTAACTTGCTATTTCTAGCTCTAAAACTTTTTTACAAATTGAGTTATCCTATAGTGAGTCGTATTA | sequence of DNA template for sgRNA transcription |
|  | sgRNAF | TAATACGACTCACTATA | amplification of DNA template |
|  | sgRNAR | CACTTTTTCAAGTTGA | amplification of DNA template |
| xCas9 expression | pETxCas9F | acctgGGTCTCACATGgataaaaagtattctattggtttagccatcggc | amplification of xCas9 gene |
|  | pETxCas9R | acctgGGTCTCAAAGCTTAgtcacccccaagctgtgaca | amplification of xCas9 gene |
|  | xCasLF | cgcaagaaccgaataCTTtacttacaagaaatttttagcaatgagatgg | C80L mutation in xCas9 |
|  | xCasLR | aatttcttgtaagtaAAGtattcggttcttgcgacgtgta | C80L mutation in xCas9 |
|  | xCasEF | tttaagaaaattgaaGAGttcgattctgtcgagatctccg | C574E mutation in xCas9 |
|  | xCasER | ctcgacagaatcgaaCTCttcaattttcttaaagtagtcctctttcaattgc | C574E mutation in xCas9 |
| SpCas9 expression | pETCas9F | acctgGGTCTCACATGGATAAGAAATACTCAATAGGCTTAG | amplification of SpCas9 gene |
|  | pETCas9R | acctgGGTCTCAAAGCTTAGTCACCTCCTAGCTGACTCAAATC | amplification of SpCas9 gene |
| pBECKP-xCas9 construction | pBECxCas9F | ggcacaagcgaaagcgcaaccccggaaagtGATAAGAAATACTCAATAGGCTTAGCTATCGG | amplification of xCas9 for pBECKP-xCas9 |
|  | pBECxCas9R | ccacaacagtacgccaaccagccaTCAGTCACCCCCAAGCTGTGA | amplification of xCas9 for pBECKP-xCas9 |
|  | pBECKPF | tcacagcttgggggtgactgaTGGCTGGTTGGCGTACTGTTGTGG | amplification of pBECKP-km backbone |
|  | pBECKPR | ccgatagctaagcctattgagtatttcttatcACTTTCCGGGGTTGCGCTTTCGCTTGTGCC | amplification of pBECKP-km backbone |
| Mutations in xCas9 | xT262AF | gaacttcgacttagctgaagatGccaaattgcagcttag | primer for xCas9 T262A mutation |
|  | xT262AR | ctaagctgcaatttggCatcttcagctaagtcgaagttc | primer for xCas9 T262A mutation |
|  | xL324RF | ccgcttcaatgatcaaacGctacgatgaacatcacca | primer for xCas9 L324R mutation |
|  | xL324RR | tggtgatgttcatcgtagCgtttgatcattgaagcgg | primer for xCas9 L324R mutation |
|  | xI409SF | ggactttcgacaacggtaGcattccacatcaaatcca | primer for xCas9 I409S mutation |
|  | xI409SR | tggatttgatgtggaatgCtaccgttgtcgaaagtcc | primer for xCas9 I409S mutation |
|  | xK480EF | gattactccatggaattttgagGAGgttgtcgataaaggtgcgtcag | primer for xCas9 K480E mutation |
|  | xK480ER | ctgacgcacctttatcgacaacCTCctcaaaattccatggagtaatc | primer for xCas9 K480E mutation |
|  | xD543EF | cgcctttctaagcggagaGcagaagaaagcaatagta | primer for xCas9 D543E mutation |
|  | xD543ER | tactattgctttcttctgCtctccgcttagaaaggcg | primer for xCas9 D543E mutation |
|  | xI694MF | cggcttcgccaataggaactttatGcagctgatccatg | primer for xCas9 I694M mutation |
|  | xI694MR | catggatcagctgCataaagttcctattggcgaagccg | primer for xCas9 I694M mutation |
|  | xV1219EF | gatgttggctagcgccggagAgcttcaaaaggggaacgaactcg | primer for xCas9 V1219E mutation |
|  | xV1219ER | tgaagcTctccggcgctagccaacatc | primer for xCas9 V1219E mutation |
| DNA oligos for crystallization | crTGGF | CAATACCATTTTTTACAAATTGAGTTAT | oligo for crystallization with TGG PAM |
|  | crTGGR | AAAATGGTATTG | oligo for crystallization with TGG PAM |
|  | crCGGF | CAATACCGTTTTTTACAAATTGAGTTAT | oligo for crystallization with CGG PAM |
|  | crCGGR | AAAACGGTATTG | oligo for crystallization with CGG PAM |
|  | crTGAF | CAATATCATTTTTTACAAATTGAGTTAT | oligo for crystallization with TGA PAM |
|  | crTGAR | AAAATGATATTG | oligo for crystallization with TGA PAM |
|  | crTGCF | CAATAGCATTTTTTACAAATTGAGTTAT | oligo for crystallization with TGC PAM |
|  | crTGCR | AAAATGCTATTG | oligo for crystallization with TGC PAM |
|  | crCGAF | CAATATCGTTTTTTACAAATTGAGTTAT | oligo for crystallization with CGA PAM |
|  | crCGAR | AAAACGATATTG | oligo for crystallization with CGA PAM |
| Construction of pUC19-spacer for in vitro cleavage assay | pUCTGGF | gatccCAATACCATTTTTTACAAATTGAGTTATg | oligo with TGG PAM |
|  | pUCTGGR | aattcATAACTCAATTTGTAAAAAATGGTATTGg | oligo with TGG PAM |
|  | pUCTGAF | gatccCAATATCATTTTTTACAAATTGAGTTATg | oligo with TGA PAM |
|  | pUCTGAR | aattcATAACTCAATTTGTAAAAAATGATATTGg | oligo with TGA PAM |
|  | pUCTGTF | gatccCAATAACATTTTTTACAAATTGAGTTATg | oligo with TGT PAM |
|  | pUCTGTR | aattcATAACTCAATTTGTAAAAAATGTTATTGg | oligo with TGT PAM |
|  | pUCTGCF | gatccCAATAGCATTTTTTACAAATTGAGTTATg | oligo with TGC PAM |
|  | pUCTGCR | aattcATAACTCAATTTGTAAAAAATGCTATTGg | oligo with TGC PAM |
|  | pUCCGTF | gatccCAATAACGTTTTTTACAAATTGAGTTATg | oligo with CGT PAM |
|  | pUCCGTR | aattcATAACTCAATTTGTAAAAAACGTTATTGg | oligo with CGT PAM |
|  | pUCAGTF | gatccCAATAACTTTTTTTACAAATTGAGTTATg | oligo with AGT PAM |
|  | pUCAGTR | aattcATAACTCAATTTGTAAAAAAAGTTATTGg | oligo with AGT PAM |
|  | pUCAGAF | gatccCAATATCTTTTTTTACAAATTGAGTTATg | oligo with AGA PAM |
|  | pUCAGAR | aattcATAACTCAATTTGTAAAAAAAGATATTGg | oligo with AGA PAM |
|  | pUCCGCF | gatccCAATAGCGTTTTTTACAAATTGAGTTATg | oligo with CGC PAM |
|  | pUCCGCR | aattcATAACTCAATTTGTAAAAAACGCTATTGg | oligo with CGC PAM |
| Editing in *K. pneumoniae* with different PAM sequences | KPTGGF | tagtTCGTCTTGAGGTTGCCGGCG | spacer for base editing in TGG site |
|  | KPTGGR | aaacCGCCGGCAACCTCAAGACGA | spacer for base editing in TGG site |
|  | KPCGTF | tagtACGATCCCGCTGGCAACAAA | spacer for base editing in CGT site |
|  | KPCGTR | aaacTTTGTTGCCAGCGGGATCGT | spacer for base editing in CGT site |
|  | KPGGTF | tagtTATCTTAACGAAGCCGCGCC | spacer for base editing in GGT site |
|  | KPGGTF | aaacGGCGCGGCTTCGTTAAGATA | spacer for base editing in GGT site |
| Editing in *K. pneumoniae* with fully matched spacers | KPsp1F | tagtCTGCTTCGCCGCTCGTCTTG | fully matched spacer 1 |
|  | KPsp1R | aaacCAAGACGAGCGGCGAAGCAG | fully matched spacer 1 |
|  | KPsp2F | tagtGGGGTCGAGGAAATAGTGCG | fully matched spacer 2 |
|  | KPsp2R | aaacCGCACTATTTCCTCGACCCC | fully matched spacer 2 |
|  | KPsp3F | tagtTCGTCTTGAGGTTGCCGGCG | fully matched spacer 3 |
|  | KPsp3R | aaacCGCCGGCAACCTCAAGACGA | fully matched spacer 3 |
|  | KPsp4F | tagtCGATGTCGCCGAGGGCGCGG | fully matched spacer 4 |
|  | KPsp4R | aaacCCGCGCCCTCGGCGACATCG | fully matched spacer 4 |
|  | KPsp5F | tagtGGTGGTCAGCGAGGCGGATA | fully matched spacer 5 |
|  | KPsp5R | aaacTATCCGCCTCGCTGACCACC | fully matched spacer 5 |
| Editing in *K. pneumoniae* with 18 and 19 mismatched spacers | KPsp1MF | tagtCgtCTTCGCCGCTCGTCTTG | 18 and 19 mismatched spacer 1 |
|  | KPsp1MR | aaacCAAGACGAGCGGCGAAGacG | 18 and 19 mismatched spacer 1 |
|  | KPsp2MF | tagtGttGTCGAGGAAATAGTGCG | 18 and 19 mismatched spacer 2 |
|  | KPsp2MR | aaacCGCACTATTTCCTCGACaaC | 18 and 19 mismatched spacer 2 |
|  | KPsp3MF | tagtTatTCTTGAGGTTGCCGGCG | 18 and 19 mismatched spacer 3 |
|  | KPsp3MR | aaacCGCCGGCAACCTCAAGAatA | 18 and 19 mismatched spacer 3 |
|  | KPsp4MF | tagtCtcTGTCGCCGAGGGCGCGG | 18 and 19 mismatched spacer 4 |
|  | KPsp4MR | aaacCCGCGCCCTCGGCGACAgaG | 18 and 19 mismatched spacer 4 |
|  | KPsp5MF | tagtGtgGGTCAGCGAGGCGGATA | 18 and 19 mismatched spacer 5 |
|  | KPsp5MR | aaacTATCCGCCTCGCTGACCcaC | 18 and 19 mismatched spacer 5 |
