# Supplementary material for: Molecular basis for the PAM expansion and fidelity enhancement of an evolved Cas9 nuclease
Source: PLoS Biol. 2019 Oct 11;17(10):e3000496. doi: 10.1371/journal.pbio.3000496 (PMC6808508; doi:10.1371/journal.pbio.3000496)
Supplement: S1 Raw Images — (PDF) [file pbio.3000496.s010.pdf]

Raw Images

Molecular basis for the PAM expansion and fidelity enhancement of an evolved Cas9 nuclease

Weizhong Chen[1], Hongyuan Zhang[1], Yifei Zhang[1], Yu Wang[1], Jianhua Gan[2]*, Quanjiang Ji[1]*

[1]School of Physical Science and Technology, ShanghaiTech University, Shanghai 201210, China.

[2]State Key Laboratory of Genetic Engineering, Collaborative Innovation Center of Genetics and Development, Shanghai Public Health Clinical Center, School of Life Sciences, Fudan University, Shanghai 200433, China.

*Corresponding author:

Quanjiang Ji: quanjiangji@shanghaitech.edu.cn

Jianhua Gan: ganjhh@fudan.edu.cn

Raw images for Fig 1A

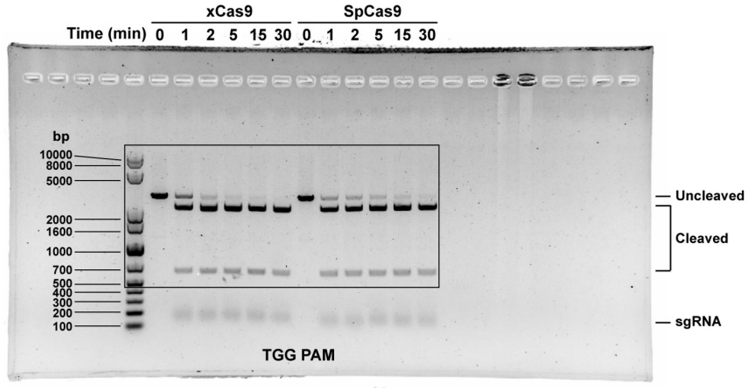

TGG PAM

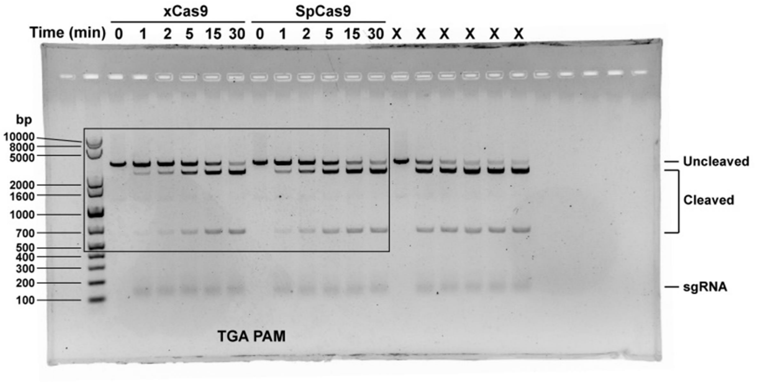

TGA PAM

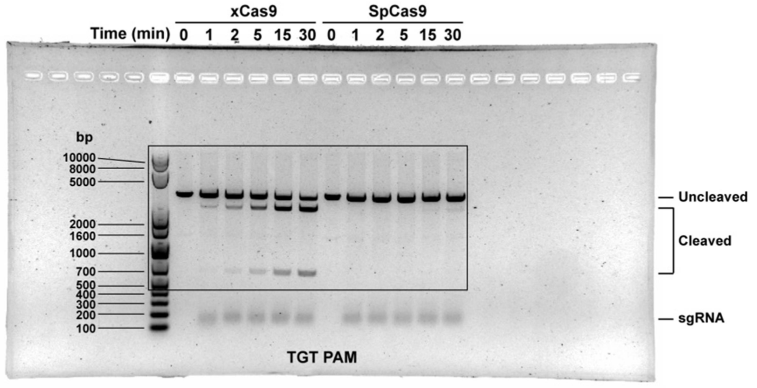

TGT PAM

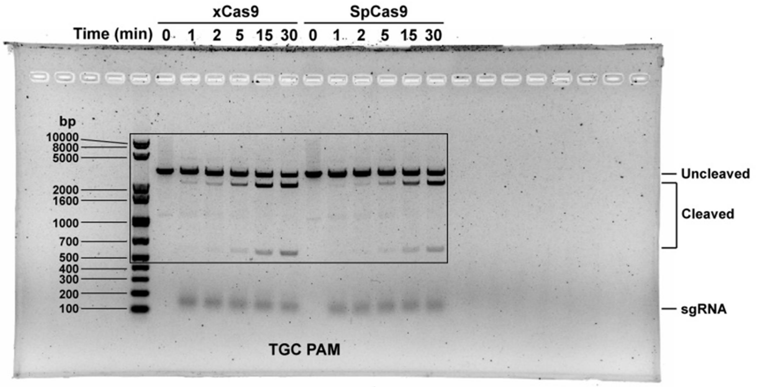

TGC PAM

Original images of agarose gel shown in Fig 1A. The area used in Fig 1A was marked with black box. The reaction solution without the addition of sgRNA was used in lanes of 0 min as control.

Raw images for Fig 6A

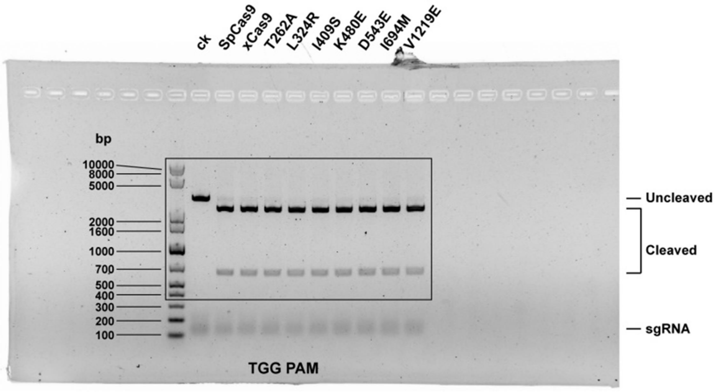

TGG PAM

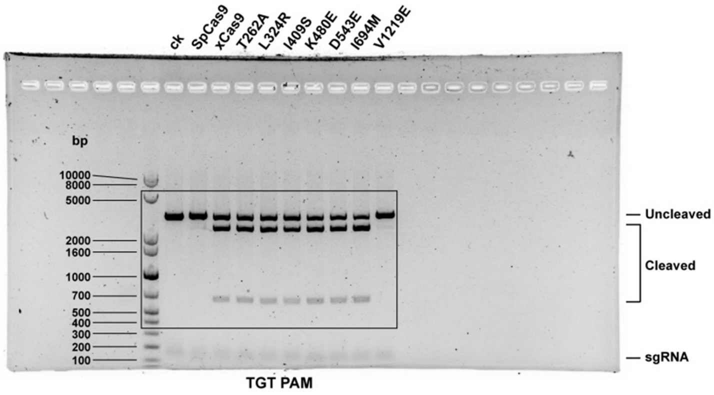

TGT PAM

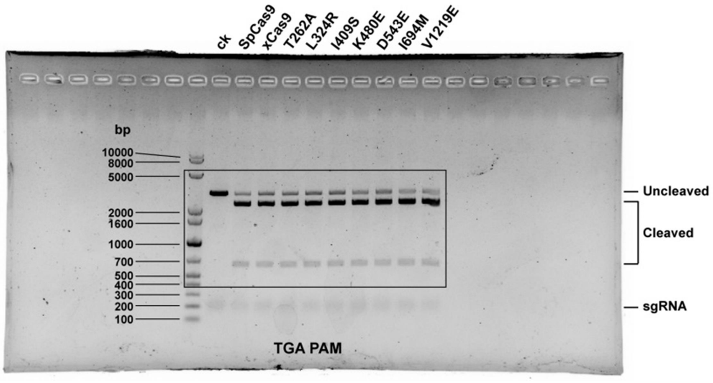

TGA PAM

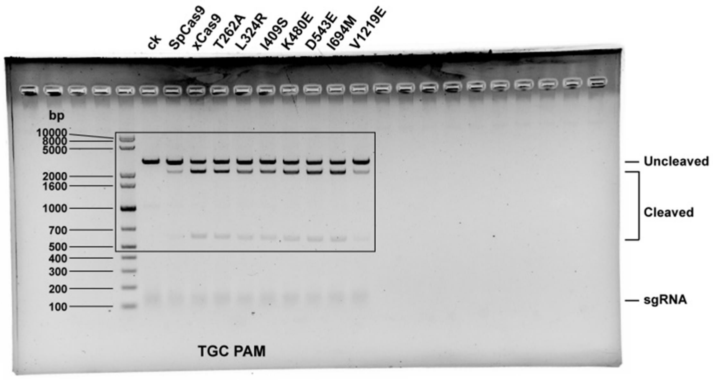

TGC PAM

Original images of agarose gel shown in Fig 6A. The area used in Fig 6A was marked with black box. The reaction solution without the addition of Cas9 protein was used in lane ck as control.

Raw images for Fig 6B

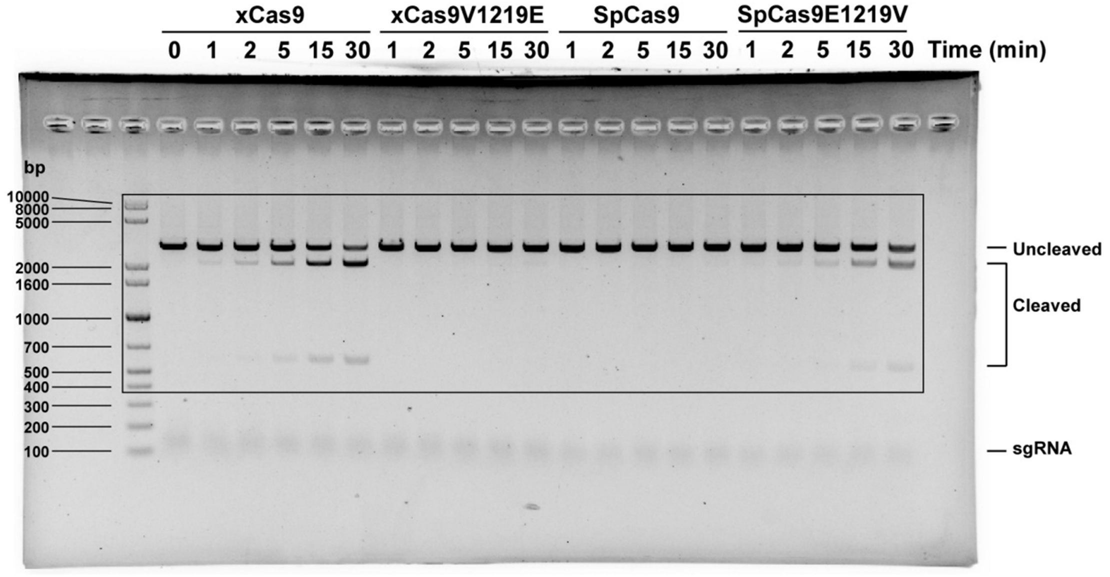

Original images of agarose gel shown in Fig 6B. The area used in Fig 6B was marked with black box. The reaction solution without the addition of Cas9 protein was used in lane of 0 min as control.

Raw images for S1 Fig.

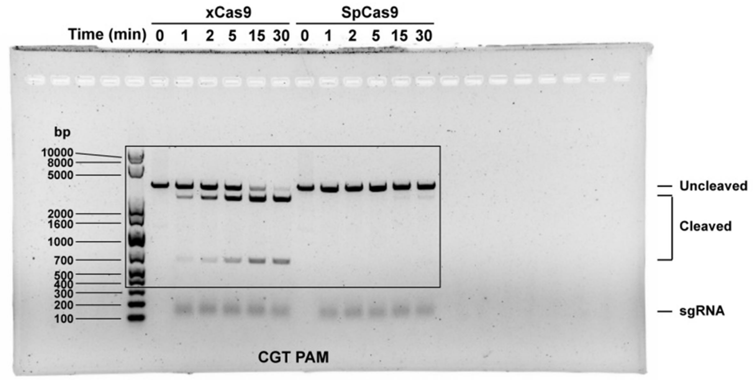

CGT PAM

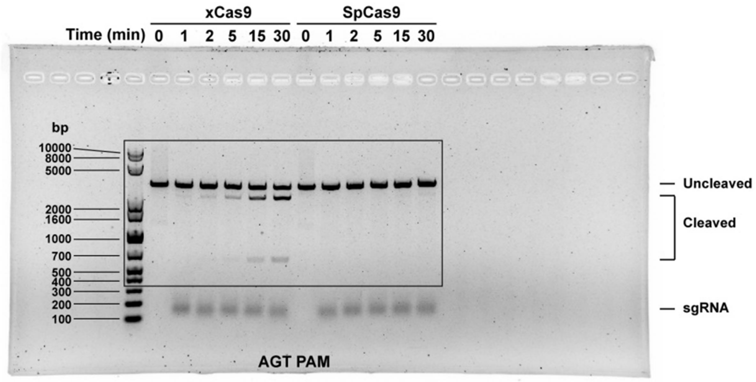

AGT PAM

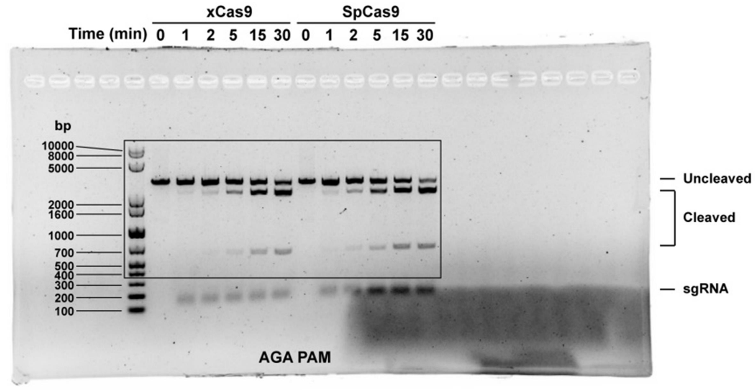

AGA PAM

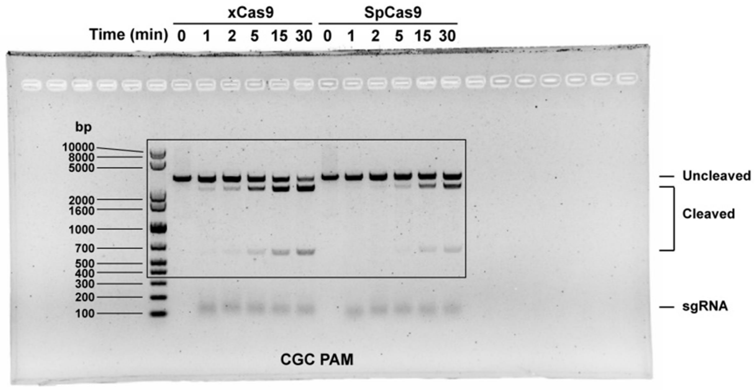

CGC PAM

Original images of agarose gel shown in S1 Fig. The area used in S1 Fig was marked with black box. The reaction solution without the addition of sgRNA was used in lanes of 0 min as control.

Raw images for S4 Fig.

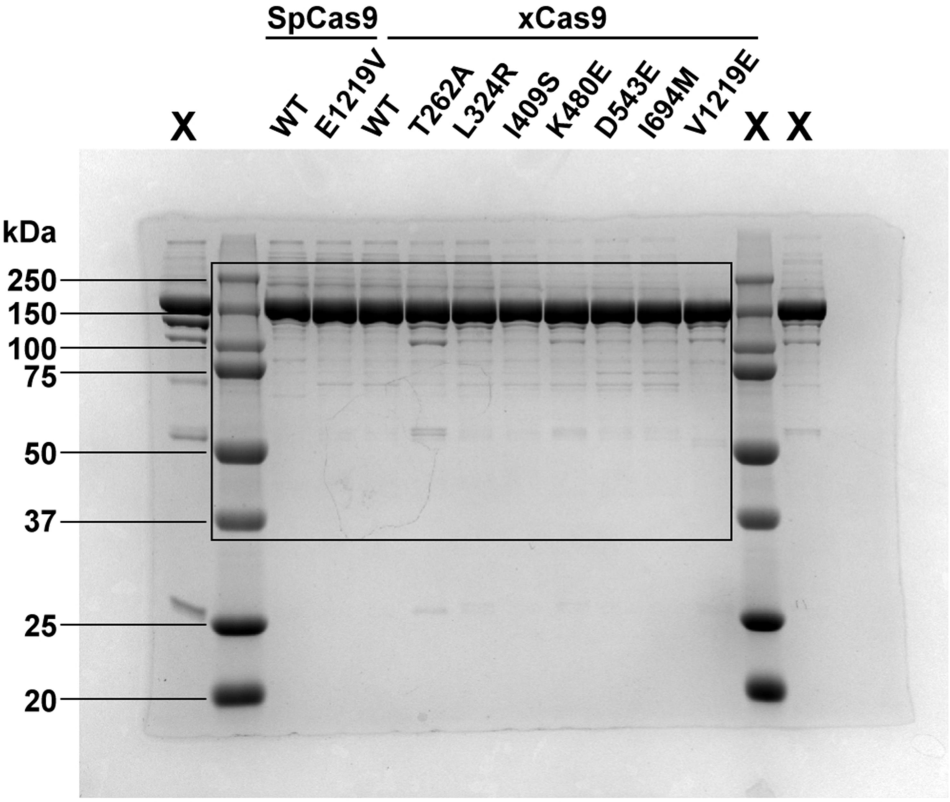

Original images of SDS-PAGE shown in S4 Fig. The area used in S4 Fig was marked with black box.
